# Hidden fairy rings and males—Genetic patterns of natural Burgundy truffle (*Tuber aestivum* Vittad.) populations reveal new insights into its life cycle

**DOI:** 10.1111/1462-2920.16131

**Published:** 2022-07-20

**Authors:** Florian Staubli, Lea Imola, Benjamin Dauphin, Virginie Molinier, Stephanie Pfister, Yasmine Piñuela, Laura Schürz, Ludger Sproll, Brian S. Steidinger, Uli Stobbe, Willy Tegel, Ulf Büntgen, Simon Egli, Martina Peter

**Affiliations:** ^1^ Swiss Federal Institute for Forest, Snow and Landscape Research WSL Birmensdorf Switzerland; ^2^ Department of Crop and Forest Sciences University of Lleida Lleida Spain; ^3^ Forest Science and Technology Centre of Catalonia Solsona Spain; ^4^ Deutsche Trüffelbäume Bodman Germany; ^5^ Department of Ecology University of Konstanz Konstanz Germany; ^6^ Chair of Forest Growth Albert‐Ludwigs University Freiburg Germany; ^7^ Department of Geography University of Cambridge Cambridge UK; ^8^ Global Change Research Centre (CzechGlobe) Brno Czech Republic; ^9^ Department of Geography, Faculty of Science Masaryk University Brno Czech Republic

## Abstract

Burgundy truffles are heterothallic ascomycetes that grow in symbiosis with trees. Despite their esteemed belowground fruitbodies, the species' complex lifecycle is still not fully understood. Here, we present the genetic patterns in three natural Burgundy truffle populations based on genotyped fruitbodies, ascospore extracts and ectomycorrhizal root tips using microsatellites and the mating‐type locus. Distinct genetic structures with high relatedness in close vicinity were found for females (forming the fruitbodies) and males (fertilizing partner as inferred from ascospore extracts), with high genotypic diversity and annual turnover of males, suggesting that ephemeral male mating partners are germinating ascospores from decaying fruitbodies. The presence of hermaphrodites and the interannual persistence of a few males suggest that persistent mycelia may sporadically also act as males. Only female or hermaphroditic individuals were detected on root tips. At one site, fruitbodies grew in a fairy ring formed by a large female individual that showed an outward growth rate of 30 cm per year, with the mycelium decaying within the ring and being fertilized by over 50 male individuals. While fairy ring structures have never been shown for truffles, the genetics of Burgundy truffle populations support a similar reproductive biology as those of other highly prized truffles.

## INTRODUCTION

The Burgundy truffle (*Tuber aestivum* Vittad.) is an ascomycetous soil fungus that forms hypogeous fruitbodies, which are coveted by chefs around the world as a culinary delicacy. As most other economically important truffle species, such as the Italian white truffle (*Tuber magnatum* Picco), the bianchetto truffle (*Tuber borchii* Vittad.) and the Périgord black truffle (*Tuber melanosporum* Vittad.), they belong to the True truffles of the genus *Tuber*. This genus contains at least 180 species, most of which are naturally distributed in the northern hemisphere and all of which live in ectomycorrhizal (ECM) symbiosis with trees and shrubs exchanging nutrients and water for sugars (Zambonelli et al., 2016). The Burgundy truffle is associated with a wide range of tree species such as oaks, beeches, hornbeams and other broad‐leaved trees, but also conifers and some shrubs such as hazels and Cistus. Occurring in a broad ecological gradient, the species is widely distributed in Europe with populations ranging from Sweden to Spain and even North Africa (Molinier et al., 2016a).

Genome sequencing of several truffle species revealed that most truffles, including *T*. *aestivum*, are heterothallic with a bipolar mating system, where a haploid individual contains a single mating‐type locus with one out of two mating‐type idiomorphs named either *MAT 1‐1* or *MAT 1‐2* (Murat et al., 2018). These encode transcription factors that are involved in the recognition of opposite mating‐type individuals and play a role during meiosis by regulating the production of pheromones and their receptors (Le Tacon et al., 2016; Rubini et al., 2011b). Haploid structures of two individuals carrying the opposite mating type have to meet for sexual reproduction and fruitbody formation. For *T*. *melanosporum*, it was shown that most parts of the fruitbody, that is the peridium and the unfertile tissues of the gleba, are formed by one of the two mating partners called the female or maternal partner and these are also found on surrounding ECM root tips, through which they obtain sugars from the host tree to grow the mycelia and nourish the fruitbodies (Le Tacon et al., 2013; Le Tacon et al., 2016). Males or paternal partners only provide genes and are detected by genotyping the ascospores that are produced within the fruitbody after meiotic recombination and by inferring their contribution to the spore genomes (Rubini et al., 2011b). An outgrowing haploid ascospore can act as a male or female, or both (called hermaphrodites), independently of the mating type it carries. Several studies reported the aggregation of *T*. *melanosporum* individuals carrying the same mating type into distinct *MAT 1‐1* and *MAT 1‐2* patches based on fruitbody and ECM root‐tip genotyping (De la Varga et al., 2017; Rubini et al., 2011a; Taschen et al., 2016). A similar aggregation of female mating types was sometimes found in *T*. *aestivum* (Molinier et al., 2016b; Splivallo et al., 2019), leading to the question of where paternal individuals of opposite mating types are located and what structure they might have (De la Varga et al., 2017; Le Tacon et al., 2016; Selosse et al., 2017; Taschen et al., 2016). Following possibilities have emerged: paternal gametes are sexual ascospores or asexual conidia, or the ephemeral hyphae germinating from these spores, or they are from mycelia that persist in soil (De la Varga et al., 2017), the first being the most likely for *T*. *melanosporum* based on spatial genetic patterns (Selosse et al., 2017).

Since *Tuber* spp. are hypogeous completing their whole life cycle hidden in the soil, they are mainly spread by small mammals and insects as spore vectors (Trappe & Claridge, 2005). They have a shorter dispersion than epigeous species, resulting in larger population differentiation and isolation by distance (IBD) (Douhan et al., 2011). Indeed, such spatial structures were shown for both *T*. *aestivum* and *T*. *melanosporum* (De la Varga et al., 2017; Molinier et al., 2016b; Murat et al., 2013; Taschen et al., 2016). To maintain a steady local population, ECM fungi need to colonize newly formed tree roots either by mycelial growth or by the recruitment of new individuals via spores. Different life strategies have been defined for plants and adopted to fungi, the simplest being the r–K model: While R‐strategists have a short lifespan and invest most of their resources into sexual reproduction and spore production, K‐strategists have a long lifespan and invest mainly in asexual mycelial growth (Douhan et al., 2011). Which strategy follows *T. aestivum* and other truffle species is not fully clear, but studies of spatial genetic patterns based on analyses of gleba (female or maternal tissue) show the presence of both, many rather small genets often forming only one fruitbody and a high genet turnover, but also a small number of individuals that persisted over several years and formed genets over 100 m in size for both *T*. *aestivum* (Molinier et al., 2016b) and *T*. *melanosporum* (De la Varga et al., 2017; Murat et al., 2013; Taschen et al., 2016).

The few previous studies addressing genetic structures of *T*. *aestivum* only focused on the distribution of maternal individuals obtained by analysing fruitbody samples. The goal of the present study was to characterize fine‐scale genetic structures of both maternal and paternal individuals in fruitbodies as well as on ECM root tips in order to gain a deeper insight in the life cycle of *T*. *aestivum* by addressing (i) the spatial distribution of mating types, (ii) the fine‐scale distribution, interannual persistence and genetic diversity of maternal and paternal individuals and (iii) the spatial correlation of *T*. *aestivum* soil mycelium amount with fruiting production. To assess this, fruitbodies were sampled in three natural *T*. *aestivum* populations located in northern Switzerland (WSL) and southern Germany (BB and Ue) over 4 (2016–2019; WSL), 3 (2011, 2013, 2019; BB) and 2 (2013, 2019; Ue) years. In two of these sites, ECM root tips were sampled and genotyped, and in one site, soil samples were collected to quantify *T*. *aestivum* mycelium.

## EXPERIMENTAL PROCEDURES

### Study range and sampling design

**FIGURE 1:**
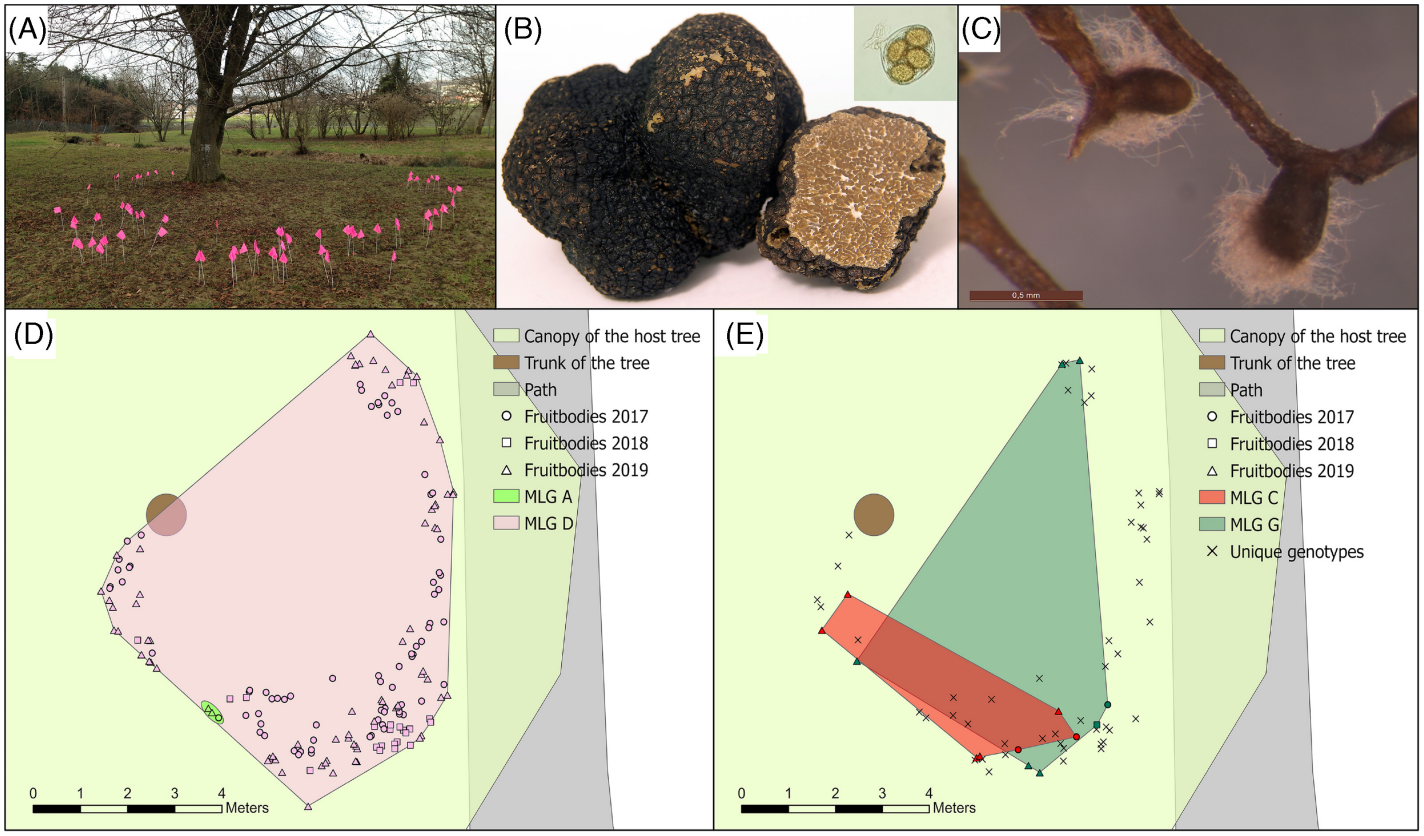
WSL Burgundy truffle sampling site with distribution of the *Tuber aestivum* multi‐locus genotypes (MLGs) identified in fruitbodies. (A) Sampling site harbouring a single *Fagus sylvatica* host tree surrounded by meadow. Positions of fruitbodies harvested in 2017 are marked with pink flags. (B) *T. aestivum* fruitbodies and ascospores (inlet). (C) Ectomycorrhizal root tips characterized by the presence of a light brown mantle and white, woolly extramatrical mycelium. (D, E) Spatial distribution of maternal (D) and paternal (E) MLGs. MLGs belonging to more than one sample are indicated by different colours and unique MLGs only present in a single sample are marked with a cross

**FIGURE 2:**
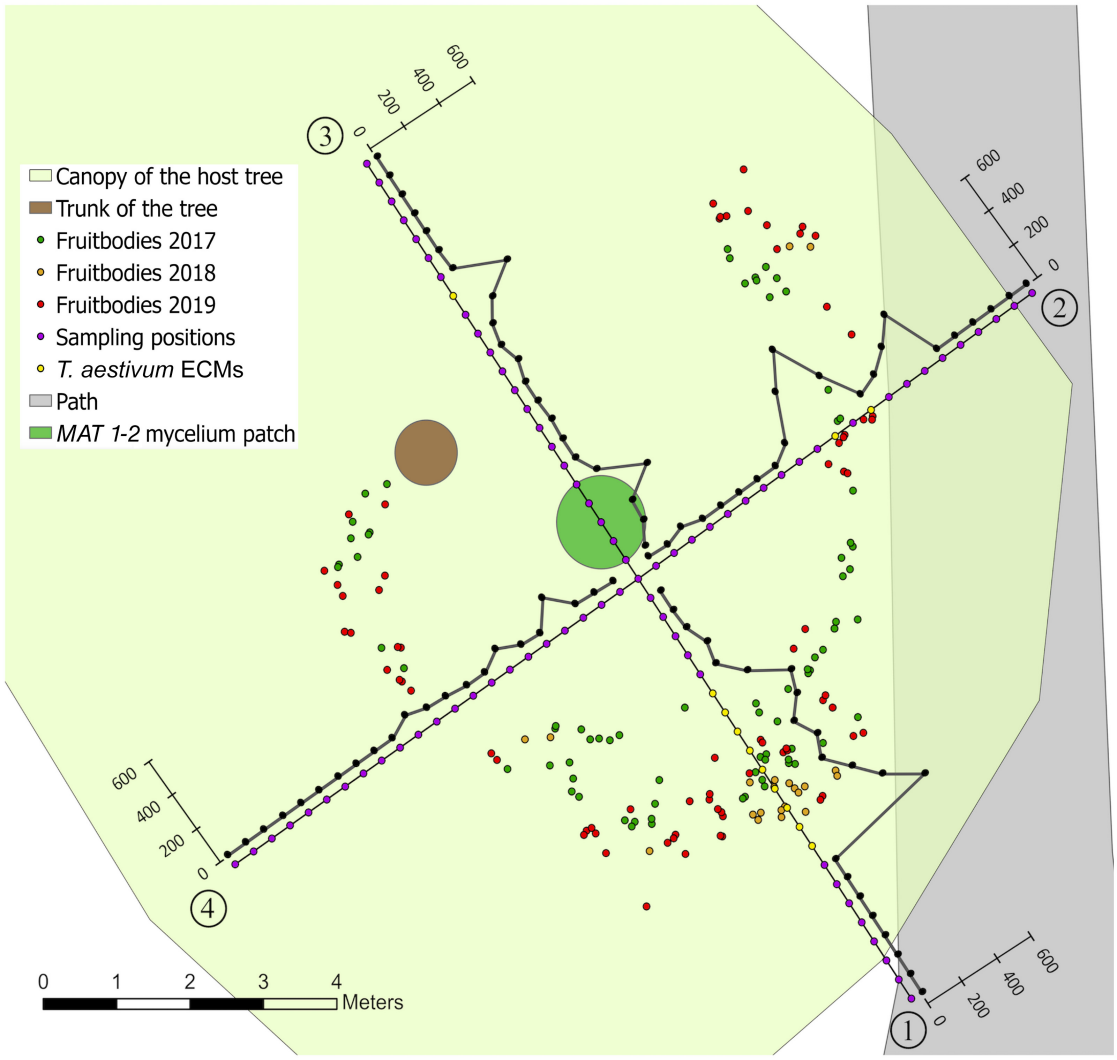
Sampling of *Tuber aestivum* fruitbodies, ectomycorrhizal root tips (ECMs) and soil samples from the WSL site. Fruitbodies sampled during different years are indicated with different colours. Four transects were defined based on the centre of the fruitbodies harvested in 2017 and 2018, on which a soil core was collected every 30 cm to gather ECMs and soil samples for mycelium quantification. Positions of ECMs genotyped as *T. aestivum* and the *MAT 1‐2* soil mycelium patch are indicated. Amounts of soil mycelium per sampling position are given in μg of dried soil mycelium per g of dried soil on the *y*‐axis of transects and are indicated by black dots

The study was conducted in three natural *T*. *aestivum* sites located in southern Germany [district of Konstanz (BB) and (Ue); Figure S1] and northern Switzerland [Birmensdorf (WSL); Figure 1]. Both sites BB and Ue are extensively described by Molinier et al. (2016b), who studied the distribution and local genetic patterns of maternal *T*. *aestivum* individuals on these sites in the years 2011 and 2013. The WSL site consists of a single isolated host tree of *Fagus sylvatica*, about 150 years old, surrounded by a fertile meadow situated within the fenced area of the Swiss Federal Research Institute WSL [Figure 2(A)].

Fruitbody material harvested by Molinier et al. (2016b) was available either dried or frozen. A new 1‐day harvest in the BB and Ue sites was conducted at the end of September 2019 with the help of four trained truffle dogs. The exact position of fruitbodies was measured with a TerraSync GPS device (Trimble International, Horgen, Switzerland). For the Ue site, we additionally included 25 fruitbodies sampled between 2016 and 2018, for which, however, no spatial localization was recorded and therefore they were not included in the main analyses but only in the comparison of genotype presence in fruitbodies versus ECM root tips. At the same site, we conducted an ECM root‐tip sampling on April 19, 2016, for which 112 soil cores with a diameter of 4.5 cm and a depth of 10 cm were taken on a regular 1.2 m grid in a rectangular study plot (14.6 m × 8.4 m; Figure S1). The samples were transferred to the laboratory and stored at 4°C until processing. The roots were cautiously washed under low running water, gathered in a 1 mm sieve and placed in a Petri dish. From each soil core, two possible *T*. *aestivum* ECMs were collected under a binocular based on morphology (Agerer, 1987‐2006; Molinier et al., 2016a) and placed individually in lysis buffer for subsequent genotyping.

For the WSL site, fruitbodies were hunted with a trained truffle dog every 3 weeks over a 4‐year time period as a part of a large‐scale monitoring project. The exact positions of fruitbodies were individually marked. Harvested fruitbodies were washed with water to remove remaining soil particles and thin slices were dried at room temperature. At the end of the three seasons (2017–2019), the georeferencing of marks was surveyed using an MS50 tachymeter (Leica Geosystems, St. Gallen, Switzerland). We determined the centre of the area in which fruitbodies were harvested in 2017 and 2018 using ArcGIS Pro v.2.4.0 (ESRI, Redlands, USA) and defined four transects with a length of 6.6 m from which soil cores with a diameter of 4.5 cm and a depth of 10 cm were collected every 30 cm in September 2019 (Figure 2). From a total of 89 cores collected, the soil was separated from the roots by carefully breaking up the soil cores, sieved with a 1 mm sieve and frozen at −18°C. In 78 cores, tree roots were present. The roots were treated as described above and inspected for *T*. *aestivum* morphotypes. An additional root sampling with eight soil cores was performed at a later stage within the patch including the *MAT 1‐2* mycelium (Figure 2) to search for ECMs of this mating type. The abundance of *T*. *aestivum* on the total number of ECMs was estimated visually. If *T*. *aestivum* was likely absent, ECMs of the most similar morphotype were sampled instead. One ECM per sampling position (and all *T*. *aestivum* morphotypes from the sampling on the *MAT 1‐2* patch) was stored in 50 μl lysis buffer PN (LGC Biosearch Technologies, Berlin, Germany) at −18°C until DNA extraction.

### DNA extraction

Genomic DNA from gleba and ECM samples were extracted using the KingFisher Flex system (Thermo Fisher Scientific, Basel, Switzerland) with the Sbeadex technology using a customized extraction protocol for plant and fungal material (LGC Genomics, Berlin, Germany). Gleba samples were disrupted using an MM400 mill (RETSCH, Haan, Germany) at 30 Hz for 2 min with a 4 mm steel ball. ECMs were lysed manually with a pestle in 50 μl lysis buffer PN (Macherey‐Nagel, Düren, Germany). After extraction, DNA was dissolved in 100 μl AMB buffer (LGC Genomics) and stored at −20°C until genotyping. ECMs sampled in 2016 were extracted using a NucleoSpin 96 Plant II Kit (Macherey‐Nagel) according to the manufacturer's instructions and DNA was eluted in 50 μl Elution Buffer PE. When using the protocols above, most of the extracted DNA results from the maternal gleba, while the ascospores remain intact. Therefore, to amplify paternal genotypes, ascospores were isolated and DNA was extracted from 99, 72 and 36 fruitbodies at the WSL, BB and Ue sites, respectively, using a protocol adapted from De la Varga et al. (2017). A small piece of dried or frozen gleba was added into a Petri dish filled with water. To release spores, the gleba surface was scraped off using either a scalpel or a pipet tip. Spores were separated from gleba pieces under the binocular using a micropipette and collected into 1.5 ml Eppendorf tubes. All centrifugation steps were conducted at 21,000*g*. Lysis was conducted with an MM400 mill (RETSCH) in 300 μl of NTE buffer (NaCl 250 mM, Tris–HCl 200 mM, EDTA 25 mM). DNA was resuspended in 100 μl of water and stored at −18°C.

To quantify *T*. *aestivum* mycelium in the soil, soil samples were lyophilized for 2 days and 250 mg was used from each sample to isolate DNA with the DNeasy PowerSoil Pro Kit (Qiagen, Stockach, Germany) according to the manufacturer's instructions. This kit proved to not extract DNA from truffle spores (Chen et al., 2021). Samples were disrupted in an MM400 mill (RETSCH) with the protocol given for the TissueLyser II and DNA was eluted in 100 μl of C6 solution and stored at −18°C.

### Molecular genotyping

Gleba, ascospore, ECM and soil samples were genotyped using the mating‐type locus and 14 nSSR markers. The mating‐type locus was amplified using the same primers and conditions as described by Molinier et al. (2016b). Reactions were performed with a final volume of 20 μl containing 0.2 μM of each primer (Microsynth, Balgach, Switzerland), 4 μg/ml of bovine serum albumin (BSA, Qiagen), 1× concentration (10 μl) of the JumpStart REDTaq ReadyMix PCR Reaction Mix (Merck KGaA, Darmstadt, Germany), 2 μl of DNA extracts (Gleba and ECM samples 1:10 diluted, ascospore and soil samples undiluted), and ultra‐pure water up to 20 μl. A total of 34 cycles for gleba, and 37 cycles for ascospore, ECM and soil samples were used. PCR products were run on a 1.5% agarose gel containing ethidium bromide for DNA labelling and visualized by using a c300 digital imager (Azure Biosystems, Dublin, USA).

Then, 14 nSSR loci (*aest01*, *aest06*, *aest07*, *aest10*, *aest15*, *aest18*, *aest24*, *aest25*, *aest26*, *aest28*, *aest29*, *aest31*, *aest35* and *aest36*) were amplified using primers developed by Molinier et al. (2013). PCRs were conducted using the Qiagen Type‐it Microsatellite PCR Kit (Qiagen). As described by Molinier et al. (2016b), two different primer mixes were used according to expected allele sizes to prevent an overlapping of fragments. Additionally, a third mix consisting of primer pairs *aest01* and *aest31* was used, since these primers show a weak amplification in the multiplex reaction. Due to a poor amplification of *aest01* and *aest31* on ascospore extractions in samples originating from southern Germany, they were excluded for downstream analysis. The monomorphic marker *aest15* was left out for the analysis of the ECM samples from Ue due to a low amplification in some samples. PCR reactions were conducted with a final volume of 10 μl containing a 1× concentration (5 μl) of the Multiplex Type‐it Reaction Mix (Qiagen), 0.2 μM primer Mix, 2 μl of Template DNA (Gleba and ECM samples 1:10 diluted, ascospore samples undiluted) and water up to 10 μl. Fluorescent forward primers were ordered at Thermo Fisher Scientific Basel and reverse primers at Microsynth AG Balgach. PCR reactions were performed using an Applied Biosystems Veriti 96 Well Thermal Cycler (Thermo Fisher) with the following conditions: 15 min at 94°C followed by 30–39 cycles of 30‐s denaturation at 94°C, 90 s of annealing at 60°C and 60 s of elongation at 72°C, with a final extension of 30 min at 60°C. Cycle number was adjusted based on previous PCR reactions. Nuclear SSR PCR products were diluted between 1:10 and 1:16 for primer mix 1 and mix 2, respectively, and 1:3 or 1:4 for primer mix 3 depending on the amplification yield of previous PCR reactions. A volume of 10 μl of the Applied Biosystems Hi‐Di Formamide (Thermo Fisher Scientific) and 0.1 μl of GeneScan 500 LIZ Size Standard (Thermo Fisher Scientific) was mixed with either 1 μl of diluted PCR products amplified with mix 1 or 1 μl of each dilution of PCR products amplified with mix 2 and mix 3. Fragment analysis was carried out in an ABI 3130 Genetic Analyser (Applied Biosystems).

### Quantification of *T*. *aestivum* soil mycelium

To quantify the amounts of soil mycelium, a real‐time qPCR approach based on the fungal ITS sequence using primers and probes designed by Gryndler et al. (2013) and a Takyon No ROX Probe Core Kit (Eurogentec, Seraing, Belgium) was used. To obtain a standard curve for absolute quantification of ITS copy numbers, a plasmid containing the *T*. *aestivum* ITS sequence was constructed as described in Supplementary Information. Reactions were performed with a volume of 20 μl containing a 1× concentration of qPCR Core Kit No ROX buffer (2 μl), 5.5 mM qPCR core kit No ROX MgCl, 0.2 mM qPCR Core Kit No ROX dNTPs, 1 U/reaction (0.2 μl) qPCR Core Kit No ROX Takyon enzyme, 0.1 mM Invitrogen Rox (Thermo Fisher Scientific), 0.2 mg/ml BSA (Qiagen), 0.3 μM *T*. *aestivum* specific primers, 0.1 μM *T*. *aestivum* specific TaqMan probe, either 5 μl of soil DNA samples (1:10 diluted) or 5 μl of plasmid DNA for the standard curve plus 5 μl of soil matrix (1:10 diluted soil extraction near the sampling site, where no *T*. *aestivum* mycelium is present) and water up to 20 μl. qPCR reactions were run in an Applied Biosystems QuantStudio 5 qPCR machine (Thermo Fisher Scientific) with the following conditions: 3 min at 95°C followed by 40 cycles of 10 s at 95°C and 60 s at 60°C. Data were analysed within the Thermo Fisher Cloud Connect by using an absolute quantification approach on triplicates of each sample. Standard curves were considered as reliable with an *R* ^2^ ≥ 0.997 and an efficiency between 90% and 110%. To assess the quality of the quantification, the coefficient of variation [(standard deviation/mean) × 100] was calculated for each triplicate. The absolute amount of soil mycelium (μg of dried mycelium/g of dried soil) was calculated based on the values obtained by Gryndler et al. (2013); 1 g of dried soil mycelium = 9.37 × 10^10^ ITS copies.

### Data analysis

#### Identification of multi‐locus genotypes

Raw nSSR data were analysed using GeneMapper v.5.0 (Thermo Fisher Scientific), applying the same bin set used by Molinier et al. (2016b), with the mating‐type locus being used as an additional marker. Only unambiguously resolved genotypes (including the mating type) were considered for the analysis. In addition, nSSR data of maternal genotypes of gleba samples previously obtained (Molinier et al., 2016b) for the sites in southern Germany for the years 2011 and 2013 were included for the analysis.

Genetic diversity [i.e. allele frequencies, unbiased expected diversity (i.e. heterozygosity in diploid data) (u*H*_e_)] and assignment of MLGs based on the nSSR profiles and the mating‐type locus were assessed for haploid and diploid (zygotes) datasets with GenAlEx v.6.512b implemented in Excel 2016 (Peakall & Smouse, 2012). To test if samples grouped into the same MLG can be considered as true clones, without carrying the same MLG by chance, the probability that copies of an MLG arose from sexual reproduction (*P*_sex_) was calculated using MLGsim with 1000 simulations (Stenberg et al., 2003). A significant *P*_sex_ value implies that copies of the same MLG are true clones and originate from asexual reproduction. Samples from MLGs having a non‐significant *P*_sex_ value were considered as unique genotypes for further analysis. Accordingly, a set of clone‐corrected MLG data were created, where the centre of each significant MLG was computed in ArcGIS Pro v.2.4.0 to overcome the bias of sampling a genotype multiple times (Taschen et al., 2016).

#### Mating type distribution

For each site, the spatial aggregation index (*Ac*) of mating types was calculated on the complete and clone‐corrected datasets for all years combined and for the year 2019 separately in the BB site using GenClone v.2.0 (Arnaud‐Haond et al., 2007). Statistical significance of the *Ac* index was tested against the null hypothesis of a spatially random distribution of samples using a re‐sampling approach based on 1000 permutations. The *Ac* index ranges from 0 to 1, where 0 indicates that the membership of nearest neighbours to the same MLG does not differ from the average probability across all occurrences, and 1 means that all nearest neighbours preferentially share the same MLG (Molinier et al., 2016b).

#### Clonal diversity and fine‐scale spatial genetic structure

Genotypic diversity was calculated as follows: *R* = (Nb. of significant MLGs − 1)/(Nb. of ramets − 1), ranging from 0 to 1, where 0 means that there is a single clone of the same MLG and 1 means that all samples belong to an individual MLG. We used GenClone v.2.0 to calculate (i) the adapted Simpson index for genotypic diversity (D*), also ranging from 0 to 1, where 1 represents maximal diversity, (ii) the Simpson evenness index (ED*), which ranges from 0 to 1, where 1 means that all MLGs show equal frequencies, and (iii) the spatial aggregation index (*Ac*) of maternal and paternal MLGs. The clonal‐specific range, defined as the farthest distance between two samples belonging to the same MLG, was measured in ArcGIS Pro v.2.4.0. For zygotes, that is fruiting bodies for which maternal and paternal genotypes were identified, inbreeding coefficients *F*_is_ (i.e. 1 − observed heterozygosity *H*_o_/mean expected heterozygosity *H*_e_) were calculated in GenAlEx v.6.512b using a diploid dataset, estimating the proportion of inbreeding under the assumption of random mating. *F*_is_ values are ranging from −1 to 1, with values close to zero representing random mating. Positive values suggest inbreeding, while negative values are an indicator of excess heterozygosity (Peakall & Smouse, 2012).

To investigate the distribution of MLGs in relation to geographic distance, we performed an IBD and a spatial autocorrelation analysis using the clone‐corrected datasets for both maternal and paternal genotypes from the two southern Germany populations. IBD analysis was conducted with the haploid data option using GenAlEx v.6.512b. Genetic and geographic distance matrices were generated and a Mantel test was performed with 999 permutations (Peakall & Smouse, 2012). To further evaluate the possible effect between genetic relatedness and geographic distances of individuals, a spatial autocorrelation approach based on the Kinship coefficient (*F*_ij_) described by Loiselle et al. (1995) was applied using SPAGeDi v.1.5 (Hardy & Vekemans, 2002). A number of 13 distance classes between 5 and 500 m were defined and it was tested whether the observed pairwise *F*_ij_ values were significantly different from 0, representing random mating, using 10,000 random permutations of spatial locations. As described by Hardy (2003), the spatial genetic structure was analysed using the *Sp* statistic, calculated as *Sp* = −*b*/(1 − *F*_1_), where *b* represents the slope of the regression of *F*_ij_ values on the natural logarithm of spatial distance and *F*_1_ is the average *F*_ij_ value of the first distance class.

#### Principal component analysis, genetic clustering and population differentiation

Principal component analysis (PCA) was conducted on a subset of the clone‐corrected dataset containing maternal and paternal genotypes originating from southern Germany and on the complete clone‐corrected dataset including all fruitbody samples from all three sites using the *dudi*.*pca* function of the R package ADE4 v. 1.7.18 (Dray & Dufour, 2007). Genotypic variation across all individuals was summarized along the three most informative axes.

Population genetic structure was investigated using Bayesian clustering analysis implemented in STRUCTURE v.2.3.4 (Pritchard et al., 2000) to determine the most likely number of genetic groups or sub‐populations. We used the clone‐corrected datasets and set the analysis with the putative populations range of *K* = 1–10, under the admixture model with correlated allele frequencies (Falush et al., 2003), and without *a priori* information of the location origin of each sample. We independently ran the analysis with 10 replicates for each value of *K*, a burn‐in of 500,000 Markov Chain Monte Carlo iterations, followed by 750,000 iterations. We inspected the convergence of the replicates and diagnosed the optimal *K* value based on the stabilization of −Log(*K*) values as visualized in Structure Harvester v.0.6.94 (Evanno et al., 2005; Earl and vonHoldt, 2012) and on the mean similarity scores computed by CLUMPAK v.1.1 (Kopelman et al., 2015). The assignment of each individual to one of the genetic clusters was based on the ancestry coefficient threshold of ≥0.5.

Pairwise genetic differences among the three populations (WSL, BB and Ue) and among the three genetic groups (sites Ue and BB) were calculated on the clone‐corrected datasets with the distance measure Φ_*PT*_ for haploid data using GenAlEx v.6.512b. In a case study of high mutation and low migration rate, Φ_*PT*_ is an appropriate estimator of genetic differentiation (Kronholm et al., 2010). Φ_*PT*_ values range between 0 and 1, where a pairwise value of 1 means two fully differentiated groups. Significance of Φ_*PT*_ values was tested using analysis of molecular variance with 999 random permutations of samples (genotypes) across the full datasets (Peakall & Smouse, 2012). We tested for spatial aggregation (*Ac* index, see above) of genetic groups in the BB site using the clone‐corrected dataset.

#### Mapping

To visualize spatial patterns, ArcGIS Pro v.2.4.0 was used to describe the sampling site and to map distribution of MLGs.

## RESULTS

### Sampling overview

In the natural *T. aestivum* site WSL, 291 *T*. *aestivum* fruitbodies were harvested over 4 years (109, 78, 28, 76; 2016–2019). Their spatial locations revealed a fairy ring structure similar to those of some epigeously fruiting fungi [Figures 1(A) and 2]. From these, we successfully genotyped 205 gleba and 66 ascospore samples. Out of 89 soil cores collected on four transects (Figure 2) to sample *T*. *aestivum* ECM morphotypes [Figure 1(C)] and to quantify mycelium, this species was present and successfully genotyped from one ECM root tip in each of 12 samples (Figure 2).

On three intensive truffle hunts with four trained truffle dogs, 74 (43 in 2013 and 31 in 2019) and 75 (33 in 2011, 39 in 2013 and 3 in 2019) fruitbodies were harvested and georeferenced in the BB and Ue sites, respectively (Figure S1). Of the sampled fruitbodies, 66 and 68 gleba, as well as 37 and 20 ascospore samples, were successfully genotyped in the BB and Ue sites, respectively. In the Ue site, we included 39 non‐georeferenced fruitbodies sampled between 2016 and 2018, of which 25 gleba and 20 ascospore samples were successfully analysed. We also collected 221 ECM root‐tip samples on a regular grid in the centre of this site in April 2016, of which 84 were genetically identified as *T*. *aestivum* and 66 could be included in the genetic analyses as all markers were successfully genotyped.

### Mating type frequency and distribution

At the WSL sampling site, all maternal gleba samples (except for one) and all ECMs were genotyped as *MAT 1‐1*. Mating type characterized on soil samples by PCR revealed that even in the soil mycelium, *MAT 1‐1* was dominating over *MAT 1‐2*. Only in 22% of the *MAT*‐PCR positive samples, both *MAT* loci were detected by non‐quantitative PCR (qPCR) analyses (Table S1). These locations did not overlap with the presence of truffle fruitbodies and usually contained low quantities of *T*. *aestivum* mycelia (Table S1). One distinct patch of *MAT 1‐2* was found at five sampling positions within the fairy ring (Figure 2). So far, no fruitbodies were harvested in this area. Since no ECM root tips of *T*. *aestivum* were present in these samples on the transect, we additionally collected eight soil cores within the mycelial patch of *MAT 1‐2*, and morphologically and genetically identified several ECM root tips as *T*. *aestivum*, which all carried the *MAT 1‐2*.

**FIGURE 3:**
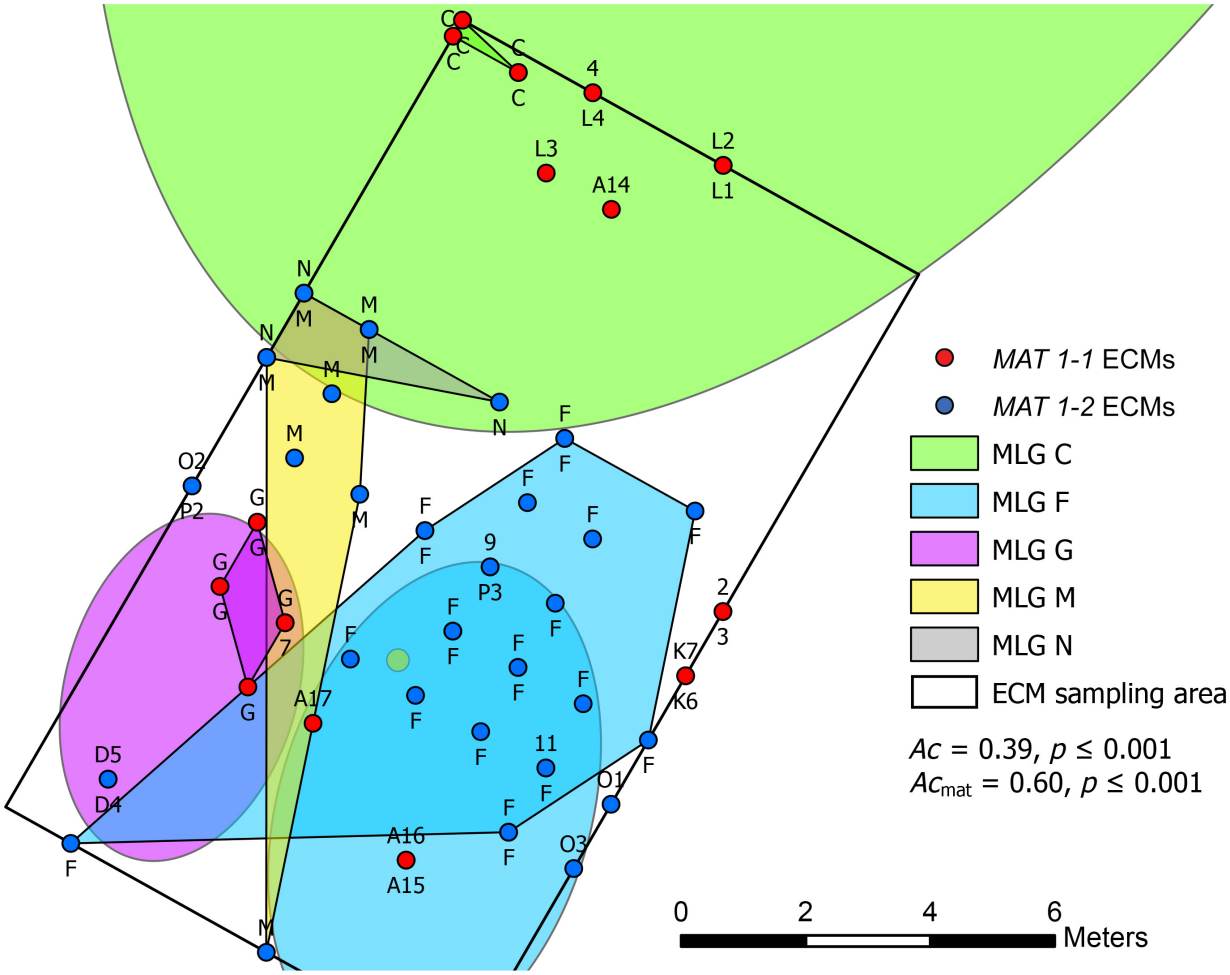
Distribution of *Tuber aestivum* multi‐locus genotypes (MLGs) identified in the ectomycorrhizal (ECM) root tips sampled in 2016 in conjunction to fruitbody MLGs. Mating types present in soil cores and the MLG membership of each ECM are given. For each sampling point, two ECM root tips of the *T. aestivum* morphotype were sampled, and in those where *T*. *aestivum* was genetically characterized, the MLG of each ECM root tip is given above and below the sampling point. MLGs consisting of multiple ECM root tips with non‐significant *P*_sex_ values are labelled with their theoretical MLG plus a number. Significant ECM MLGs are indicated by polygons and the spatial location of their corresponding fruitbody MLG by ellipses in the same colour. The aggregation (*Ac*) of ECM MLGs was calculated with all ECM samples. The mating‐type aggregation index *Ac*_mat_ was calculated using only one ECM sample per soil core (both ECM root tips always showed the same mating type) and corrected for clones

In the sites in southern Germany, mating types were significantly aggregated for both the complete and clone‐corrected datasets in the Ue site (*Ac* = 0.74 with *p* = 0.001 and *Ac* = 0.47 with *p* = 0.02, respectively) and for the complete (*Ac* = 0.52, *p* < 0.001), but not the clone‐corrected dataset (*Ac* = 0.31, *p* = 0.06; Table 1) in the BB site. A less pronounced aggregation of mating types in the BB than the Ue site can be observed when visualized on maps (Figure S2). In the Ue site, the aggregation of mating types was also present at the root level on ECMs (*Ac* = 0.60, *p* < 0.001; Figure 3). Within the same soil core, the two randomly selected *T*. *aestivum* morphotypes always showed the same mating type.

**TABLE 1:** Genotypic diversity parameters of the natural *Tuber aestivum* populations

Samples	Nb. of samples (*N*)	Nb. of MLGs (*G*)	Genotypic richness (*R*)	Simpson's diversity index (D*)	Evenness (ED*)	Genetic diversity (u*H*_e_)	Inbreeding coefficient (*F*_is_)	Aggregation index Ac (*p*‐value)	Clonal subrange (m)	MAT aggregation index Ac complete dataset (*p*‐value)	MAT aggregation index Ac clone‐corrected dataset (*p*‐value)
WSL maternal	205	2	0.005	0.048	0.077		–	1.000 (0.00)	9.69	–	–
WSL paternal	66	54	0.803	0.793	0.672		–	0.169 (0.01)	8.42	–	–
WSL combined	271	56	0.204	0.444	0.377	0.09	−0.026	–	9.69	–	–
BB maternal	66	34	0.508	0.893	0.716		–	0.372 (0.00)	164.09	0.52 (0.00)	0.31 (0.06)
BB paternal	37	31	0.833	0.971	0.808		–	−0.002 (0.56)	154.33	–	–
BB combined	103	63	0.608	0.932	0.769	0.40	0.232	–	185.9	–	–
Ue maternal	68	33	0.478	0.878	0.905		–	0.715 (0.00)	45.48	0.74 (0.00)	0.47 (0.02)
Ue paternal	20	20	1.000	0.932	0.479		–	0.088 (0.19)	0	–	–
Ue combined	88	51	0.575	0.911	0.907	0.19	0.172	–	45.48	–	–

Results are shown for maternal (gleba) and paternal (ascospores) datasets separately, and a combined dataset containing both maternal and paternal samples. Genotypic richness, Simpson's diversity index and evenness is based on genotypic data, while genetic diversity is based on allele number and frequencies and represents the mean value over all loci of the clone‐corrected dataset.

### Analysis of multi‐locus genotypes and genetic diversity

All nuclear simple sequence repeats (nSSR) markers were polymorphic across all sites, except for *aest15* (Table S2). The number of alleles per locus ranged from one to eight, with five loci being monomorphic for the WSL and Ue sites, and one for the BB site. Overall, low values of effective allele and unbiased expected diversity were found; with the mean u*H*_e_ = 0.06/0.09, 0.19/0.19 and 0.33/0.40 (all samples/clone‐corrected) at the WSL, Ue and BB site, respectively (Table 1, Table S2).

In fruitbodies, 56 multi‐locus genotypes (MLGs) were detected at WSL (i.e. two maternal, 54 paternal), 63 in the BB (i.e. 32 maternal, 29 paternal and two hermaphrodites) and 53 in the Ue site (i.e., 31 maternal, 18 paternal and two hermaphrodites; Tables S3 and S4). Of the MLGs found in multiple samples, which are true clones as they showed a significant *P*_sex_ value, all at WSL (two maternal and two paternal) and Ue (two maternal and two hermaphrodites) and one hermaphrodite out of the four MLGs (two maternal and two hermaphrodites) at the BB site were present over several years (Table S3). One of these MLGs persisted for 9 years (i.e. 2011–2019; a maternal individual in the Ue site). Hermaphroditic MLGs with significant *P*_sex_ values made up 3.17% and 3.92% of the total number of MLGs in the BB and Ue site, respectively. While paternally acting ramets of these MLGs were found in only 1 year, maternally acting ones were detected over several years for three of the four hermaphrodites (Table S3). No MLG was shared between the three analysed sites.

All ECMs of *T*. *aestivum* sampled along the transects at the WSL site showed the same genetic profile as the dominant maternal individual. However, the additional five ECM root‐tips sampled within the *MAT 1‐2* mycelial patch and characterized as such, revealed a new MLG (*P*_sex_ = 0.02, *p* = 0.04) showing a new allele for the locus *aest18*, which so far was monomorphic for this site. All other nSSR markers of this new MLG showed the same allele as the dominant maternal individual.

At the Ue site, comparing MLGs present in fruitbodies (including additional fruitbodies from the years 2016–2018) and ECMs collected in 2016, 68 MLGs were exclusively detected in fruitbodies, 21 only in ECMs, and six in both (considering non‐significant MLGs as multiple individuals; Table S3). For those MLGs that persisted over the years and were true clones, all but two (MLG I and MLG Q, both located over 36 m away from the ECM sampling plot) were identified in ECMs. With this enlarged dataset, we identified three additional perennial MLGs, including one hermaphrodite (MLG P), which acted paternally in 2011, maternally in 2016 and 2017 and was present in two ECM root tips in 2016. MLG D, which only occurred twice in paternal ascospore samples in 2011 and 2013, was also detected in the ECMs sampled in 2016, but this finding requires further investigations given the non‐significant *P*_sex_ value, meaning that MLG D cannot be considered as a true clone. Therefore, only maternal and hermaphroditic genotypes were present at ECM root tips.

### Genotypic diversity and spatial genetic structure at the WSL site

Genotypic diversity and evenness at the WSL site was rather low (*R* = 0.201, D* = 0.444, ED* = 0.377; Table 1). Only two maternal MLGs were observed compared to the 54 paternal ones, with maternal MLG D showing a frequency of 0.976 [Figure 1(D,E); Table S3]. This is also represented in the genotypic diversity being much lower for females (*R* = 0.005, D* = 0.048) than for males (*R* = 0.785, D* = 0.793) and higher aggregation of females (*Ac* = 1.000, *p* < 0.001) compared to males (*Ac* = 0.169, *p* = 0.01; Table 1). Of the 54 paternal individuals, only two were found in several samples and persisted over the years. One of them exhibited a similar clonal subrange (8.4 m) as the dominating maternal individual (9.7 m; Figure 1(D,E); Table 1). The inbreeding coefficient (*F*_is_) calculated for zygotes, that is fruitbodies for which maternal and paternal alleles are available, was negative (*F*_is_ = −0.026), indicating random mating within this site.

### Genotypic diversity and spatial genetic structure in southern Germany

High genotypic diversity was found in both sites, BB (*R* = 0.608, D* = 0.932) and Ue (*R* = 0.575, D* = 0.911). As at the WSL site, paternal diversity was higher than maternal diversity (Table 1). Maternal but not paternal MLGs were significantly aggregated, more pronounced in the Ue (*Ac* = 0.715, *p* < 0.001) than in the BB (*Ac* = 0.3721, *p* < 0.001) site (Table 1; Figure S3). A maximum clonal subrange of 185.9 m was measured in BB, an MLG acting as hermaphrodite (MLG E), and 45.5 m in Ue, a maternally acting MLG (MLG C; Table 1, Table S3). At the Ue site, all paternal individuals were unique MLGs. The distribution of maternally and paternally acting ramets within hermaphrodites shows visually no clear spatial pattern. Paternal ramets were more scattered, while maternal ones show a stronger aggregation for MLG D, but the opposite was found for MLG E in the BB site (Figure S4).

When looking at the clone‐corrected datasets, significant IBD was observed for both maternal (*R* = 0.422, *p* ≤ 0.001) and paternal (*R* = 0.385, *p* ≤ 0.007) individuals in the Ue, but only for paternal ones in the BB site (*R* = 0.208, *p* = 0.036; Figure S5). In general, a weaker IBD was assessed in the BB than in the Ue site. At the Ue site, both maternal and paternal genotypes showed a similar *R*‐value supporting similar dispersion capacities (Figure S5). Spatial autocorrelation evaluated with the Kinship coefficient (*F*_ij_) was significant up to 6 m for maternal and paternal individuals in both BB and Ue sites (Figure S6). *Sp* values indicating the decrease of pairwise kinship over geographic distance were slightly higher for both maternal and paternal individuals in the Ue (maternal: *Sp* = 0.13, paternal: *Sp* = 0.08; Figure S6) compared to the BB (maternal: *Sp* = 0.08, paternal: *Sp* = 0.07) site, coinciding with the IBD analysis (Figure S5). Considering only the first 5 m for the regression analysis, both sites showed similar *Sp* values with high estimates for maternal (*Sp* = 0.55 and 0.54 for the BB and Ue site, respectively) and low estimates for paternal individuals (*Sp* = 0.05 for both BB and Ue sites), suggesting a strong genetic structure within the first 5 m for females, but not males (Figure S6). Looking at the zygotes, high inbreeding was observed for both BB (*F*_is_ = 0.232) and Ue (*F*_is_ = 0.172; Table 1) sites.

#### Population differentiation and subdivision

**FIGURE 4:**
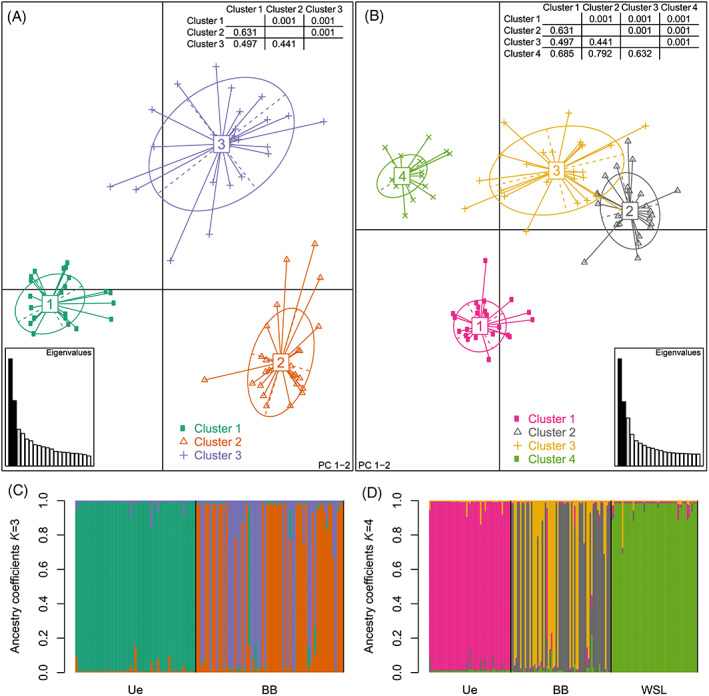
Genetic structure of *Tuber aestivum* populations based on the subset (only populations in Southern Germany) and complete clone‐corrected dataset. (A, B) Principal component (PC) analyses on the clone‐corrected subset containing samples from BB and Ue (A) and on the complete clone‐corrected dataset consisting of samples from all three populations (B). Pairwise Φ_*PT*_ values, representing the genetic differentiation between groups, are given in the lower half of the matrices with corresponding *p* values from the AMOVA analysis in upper half (inlet tables). (C, D) Admixture analysis among the two (C) and three (D) populations of *T*. *aestivum* based on the clone‐corrected datasets. Each barplot shows the level of admixture within individuals and colours reflect assignment probabilities to respective genetic clusters. The assignment of each individual to one of the genetic clusters was based on the ancestry coefficient threshold of ≥0.5 (Table S4). AMOVA, analysis of molecular variance

For the two sites in southern Germany, we found three genetic clusters with high genotypic variation and low levels of admixture within individuals [Figure 4(A,C), Figures S7, S9, S11]. All individuals in the Ue site were assigned to one cluster, whereas within the BB site, two main genetic clusters were characterized [cluster 1 in Ue, clusters 2 and 3 in BB; Figures 4(C), Table S4]. However, one unique maternal MLG, found in the BB site in 2019, was grouped into the genetic cluster 1 which dominated in the Ue site [Figure 4(C), Table S4]. The three clusters were strongly differentiated as indicated by high Φ_*PT*_ values [Figure 4(A)] and were significantly spatially aggregated in the BB site considering maternal MLGs (*Ac* = 0.43, *p* = 0.009) but not for paternal ones (*Ac* = 0.37, *p* = 0.065; Figure S2). In 92% of the 37 zygotes analysed in the BB site, maternal and paternal MLGs shared the similar cluster memberships and only three fruitbodies arose from mating of individuals from different genetic clusters (clusters 2 and 3; Figure S2).

The three populations BB, Ue and WSL were clearly differentiated based on principal component and Bayesian clustering analyses [Figure 4(B,D), Figure S8]. We inspected the estimated probability distribution across *K* values and found at *K* = 4 a clear separation of the three populations and a substructure in the BB site as supported by the PCA analysis (Figures S10 and S12). The four genetic clusters differed by high Φ_*PT*_ values, with the lowest value found between clusters 2 and 3 in the BB site [Figure 4(B)].

### Hidden fairy ring

Fruitbodies at the WSL site grew on a fairy ring structure with a radial outgrowth trend of around 30 cm per year (Figure 2). Mycelium quantifications and ECM assessment along the four transects revealed a high correlation between the amounts of soil mycelium, the presence of *T*. *aestivum* ECMs and the production of fruitbodies on the ring (Figure 2). When *T*. *aestivum* ECMs were present, they usually colonized a high percentage of the fine roots in that sample (up to 80%). Soil mycelium of *T*. *aestivum* ranged from 0 μg dried mycelium per gram dried soil to a maximum of 592.7 μg g^−1^ soil (Table S1). The highest amounts of soil mycelium were detected on transect 1, where also many fruitbodies were produced and many ECMs were found. A peak of soil mycelium was detected on the inside of the ring on transect 3 spanning over five sampling positions, which was genotyped as *MAT 1‐2* (Figure 2).

## DISCUSSION

### Mating type distribution

Like other *Tuber* species, *T*. *aestivum* is a heterothallic fungus with a dominant haploid life cycle, where two haploid mycelia from opposite mating type have to meet for sexual reproduction (Molinier et al., 2016a). Several studies of Périgord truffle (*T*. *melanosporum*) populations revealed a strong mating‐type aggregation with spatial structuring of maternal individuals carrying the opposite *MAT* locus (De la Varga et al., 2017; Murat et al., 2013; Rubini et al., 2011a; Taschen et al., 2016). The same was recently described in a *T*. *borchii* plantation (Leonardi et al., 2019). Such aggregation of mating types seems to occur in the Burgundy truffle as well, but at a lesser prevalence with mixed patterns based on current and previous studies. Splivallo et al. (2019) analysed the distribution of mating types in two *T*. *aestivum* orchards, where in one orchard the mating types were uniformly distributed and in the other a strong aggregation of *MAT 1‐1* individuals was found. In our study, aggregation was observed in the BB site and occurred significantly in the Ue site, not only as revealed by the fruitbody data but also as evidenced by the ECM root level and soil mycelia in the WSL site. Rubini et al. (2011a) suggested that spatial structuring of mating types could be due to competitive exclusion between different genotypes, in which the mating‐type locus is involved as a marker of self‐recognition and vegetative incompatibility, as shown for other fungal species [i.e. *Neurospora crassa*, *Sordaria brevicollis*, *Ascobolus stercorarius* and *Aspergillus heterothallicus* (Glass et al., 2000)]. However, for sexual reproduction, tissues of opposite mating type have to fuse, which suggests that incompatibility is suppressed in reproductive structures. How this is accomplished is unclear, but studies on *N*. *crassa* indicate that repressed transcription of loci mediating mating‐type associated vegetative incompatibility might be the mechanism (e.g. *tol* locus; Shiu & Glass, 1999). Under the assumption that sexual reproduction is costly and controlled by a trade‐off, mating‐type associated vegetative incompatibility could prevent too frequent mating due to the low probability that two compatible individuals of opposite mating type meet (Selosse et al., 2013). Further research is needed to demonstrate whether such a mechanism exists and could be activated or inhibited depending on unknown intrinsic or extrinsic factors.

### Genetic diversity and spatial genetic structure

Our sites revealed low genetic diversity with usually one dominant allele at a given locus, especially in the WSL and Ue site (u*H*_e_ = 0.09 and 0.19, respectively), although the markers were established as highly polymorphic (Molinier et al., 2013). This is likely due to the geographical range of our study being restricted to a regional scale. The few studies that reported genetic diversity using similar numbers of nSSR markers showed generally higher values of expected diversity for natural *T*. *aestivum* (*H*_e_ = 0.31–0.62; Molinier et al., 2013; Molinier et al., 2015a; Molinier et al., 2015b) and for natural and planted *T*. *melanosporum* populations (*H*_e_ = 0.21–0.59; Taschen et al., 2016; De la Varga et al., 2017) compared to our findings. The two sites WSL and Ue are relatively isolated and most likely the gene pool is rarely enriched by migrants. Although genetic diversity was low, genotypic diversity was high and similar to what is found in other truffle sites (e.g. 0.04–0.64 maternal, 0.50–1.00 paternal; Molinier et al., 2015a; Taschen et al., 2016; De la Varga et al., 2017; Schneider‐Maunoury et al., 2020). This also indicates that several closely related individuals have established these populations. Consistent with the high prevalence of non‐significant *P*_sex_ values in all sites, more nSSR markers or next‐generation sequencing data would have been needed to discriminate all individuals in these populations at a deeper resolution. Furthermore, at sites in southern Germany, we found high inbreeding coefficients, suggesting that maternal and paternal gametes of zygotes are closely related to each other. Similar *P*_sex_ values and higher inbreeding coefficients than our measurements were reported in several natural and planted *T*. *melanosporum* populations using 10–13 nSSR markers (De la Varga et al., 2017; Riccioni et al., 2008; Schneider‐Maunoury, 2019; Taschen et al., 2016). Inbreeding was also indicated for *T*. *magnatum* (Paolocci et al., 2006) but not for *T*. *borchii*, the only *Tuber* species of these four for which conidia formation has been reported (Leonardi et al., 2019). At the WSL site, the whole population is probably composed of closely related individuals and therefore, neither inbreeding (which in principle determines whether closely related individuals mate more often than expected by chance within this population) nor a clear spatial structuring was detected due to lack of genetic variability. For the sites in Germany, a strong spatial structuring of males and females indicates that closely related individuals are spatially close and reproduce sexually. The same pattern was observed for *T*. *melanosporum* and might arise under the following conditions, as hypothesized by Selosse et al. (2017): (i) spores originating from one or a few fruitbodies are locally deposited by animals, so that these immigrated spores, which will grow into maternally and paternally acting gametes, are genetically similar; (ii) male gametes are seldom dispersed; (iii) since IBD was also observed in truffle plantations, where plants have been inoculated with several fruitbodies and spore mixtures are additionally disseminated in the field, other mechanisms besides the local deposition of a few genetically related spores must drive such spatial genetic patterns. Selosse et al. (2017) hypothesized that vegetative incompatibility is not exclusively controlled by loci close to the mating type or the mating type itself, but also by loci in other parts of the genome. Interestingly, our autocorrelation analyses at sites in Germany showed that at very small spatial distances (i.e. <5 m), maternal individuals have a much stronger genetic structure than paternal ones. This pattern could be explained by such a vegetative incompatibility mechanism, translated into competition effects, which may act more strongly on maternal individuals forming longer‐lived mycelia and ECM root tips than on ephemeral paternal structures.

### Colonization strategies, hermaphrodites and genets at ectomycorrhizal root tips

Our observations on the presence, persistence and turnover of maternal, paternal and hermaphroditic genotypes in the three sites are consistent with what has been found for *T*. *melanosporum*, confirming that (i) *T*. *aestivum* is hermaphrodite with usually forced unisexual behaviour with paternal, but also many maternal genotypes being short‐lived and generally propagating over short distances by sexual spores (ii) a few, but often dominating maternal individuals spread by vegetative growth and produce most of the fruitbodies, in our case more than half of all truffles in each of the sites and (iii) hermaphroditism is possible but rare, in our case only 3%–4% of all characterized genotypes, as similarly found for the Périgord truffle (De la Varga et al., 2017; Schneider‐Maunoury, 2019; Selosse et al., 2017; Taschen et al., 2016). At each site, one to four maternal genotypes produced fruitbodies over several years, up to 9 years in the case of the Ue site. Two hermaphrodites at the BB site spread up to 185 m, which is in the same range as found for the Périgord truffle in a natural truffle ground in southern France (Taschen et al., 2016). If these genotypes had spread only by vegetative growth, then *T*. *aestivum* would be one of the largest genet forming ECM fungus (Douhan et al., 2011).

For the WSL site, all ECM root tips analysed on the transects showed the same genotype as the large maternal individual producing the fruitbodies, which further supports that *T*. *aestivum* has a life cycle similar to the one proposed for *T*. *melanosporum*, with only maternal individuals being present at ECM root tips (Murat et al., 2013; Rubini et al., 2011a; Schneider‐Maunoury et al., 2020; Selosse et al., 2017). A substantial spatial overlap of maternal or hermaphroditic fruitbody MLGs with the occurrence of belowground ECM was also found at the Ue site, although many individuals were exclusively detected either in fruitbodies or in ECMs. In the first case, this was expected as we only sampled ECMs once in 2016 in a restricted area but have fruitbody occurrences from 2011 to 2019. In the second case, this may indicate that not all MLGs being present at root tips produce fruitbodies every year, as reported for *T*. *melanosporum* (De la Varga et al., 2017) and other ECM fungal species (Douhan et al., 2011), or that we simply missed them during our sampling campaigns. Furthermore, we found only maternal or hermaphroditic MLGs on ECMs and no individuals acting solely paternally.

While maternal individuals of fruitbodies stem from the same mycelium forming the ECM root tips, the origin of paternal genotypes is still unknown. High genotypic variation and annual turnover of males observed in the present study support the hypothesis that paternal genotypes are recruited from ascospores, where a spore originating from a decomposed fruitbody, or spread by an animal, germinates and mates immediately without the formation of a persistent mycelium. This is also indicated by the observed aggregation of mating types from maternal individuals, which reduces intermingling and the probability that two persistent mycelia of opposite mating type meet. In addition, De la Varga et al. (2017) reported that for *T*. *melanosporum*, most fruitbodies are formed within a cluster of mating types and not at the boundary between them where mycelia would meet, which is likewise spatially explicit in our study case. These findings bear major implications for truffle farming, because they suggest that the application of ascospores on and in soil such as in traps (Murat et al., 2016) is a scientifically founded practice to promote fruiting by providing male partners for fruitbody formation. In fact, this has been practised for some time by truffle farmers based on their successful experience (De la Varga et al., 2017).

If we assume that hyphae outgrowing from ascospores function as male gametes, then we should principally be able to find both mating types as mycelia in the soil, at least in certain time periods. Chen et al. (2021) in fact reported the detection of both mating types in 38% and 77% of soil samples under non‐productive and productive trees, respectively, in *T*. *melanosporum* plantations in France. They suggested that recording the presence of both mating types could be used as a tool to optimize the truffle orchard management. In our case, we never detected both mating types in the soil where truffles were produced at the WSL site, but only in a few samples that revealed small amounts of truffle mycelia. Although we used less sensitive methods than Chen et al. (2021), which might explain this discrepancy, the time point at which these samples were taken may also be critical, assuming short‐lived paternal mycelia. In a *T. aestivum* plantation in France, the lowest amounts of *T. aestivum* mycelia were found in March/April and the highest tended to be in July/August and December (Todesco et al., 2019). So the latter might be good periods to check for the presence of both mating types in the same soil core (we took them in September). Less common, persisting mycelia also produce male gametes as indicated by our data and in previous reports (De la Varga et al., 2017; Schneider‐Maunoury et al., 2020). This is the case when hermaphroditic genotypes are present at ECM root tips and act maternally and paternally as identified in southern Germany. However, at the WSL site, two paternal individuals must have persisted for several years by acting exclusively paternally and without being detected on ECM root tips. It is unclear what ecological niche these individuals occupy and how they can survive without a carbon supply of an autotrophic partner. It is unlikely that they can persist as free‐living soil mycelium for several years, since the sequencing of *Tuber* spp., including *T*. *aestivum*, revealed a high reduction of plant cell‐wall degrading enzymes (PCWDEs) which would be required for a saprophytic lifestyle (Murat et al., 2018). However, note that few PCWDEs were kept and the enzymes for fungal cell‐wall degradation are not reduced, which might allow carbon to be gained from fungal and to some extent plant necromass (Miyauchi et al., 2020). A more intensive search for ECMs of paternal genotypes should reveal whether a few ECM root tips might be sufficient for paternal survival.

#### Restricted gene flow within and among populations

In southern Germany, we found that gene flow between the sites seems to exist but is relatively rare based on well‐differentiated genetic clusters, even if they are only 3 km apart. Even within the BB site, high pairwise Φ_*PT*_ values between the genetic clusters imply low gene flow leading to well‐differentiated subpopulations. These subpopulations are spatially structured, even over the sampling years (e.g. 2013 and 2019). At locations where the clusters spatially overlapped, we found three fruitbodies that arose by mating of individuals belonging to two different clusters. This indicates that no intrinsic genetic barrier exists and gene flow is possible, but rarely happens, most likely because the clusters are spatially separated. This was similarly concluded for *T*. *melanosporum*, where mating of unrelated genotypes was occasionally found in zygotes (De la Varga et al., 2017), but mostly, closely related spores released from decaying fruitbodies fertilize their ‘mother’ individual (which initially formed the fruitbody) because they are sufficiently close in space. In other words, this very local fertilization favoured by limited spore dispersal ability entails a non‐random sorting of alleles within populations, which results in high genetic structuring within a site. Such restricted gene flow might be advantageous for local adaptation as long as the individuals carry beneficial allele(s), but it is unclear whether it is critical for adaptation to rapid environmental changes as this may depend on previous stressful environmental conditions (Reed et al., 2003).

### Hidden fairy ring at WSL

To our surprise, the fruitbodies at the WSL site were growing in a fairy ring structure in the rooting zone of the host tree *Fagus sylvatica*, the ring being shifted in relation to the trunk of the tree. To our knowledge, this is the first time such a structure is described for a truffle species. Fairy rings, also called witch rings, are fungal fruitbodies growing in a circular structure in grasslands or around a host tree and consist of one or several individuals whose mycelia grow radially out from a central point (Peter, 2006). In our study, the mycelium was formed by one large individual as indicated by the nSSR data of maternal gleba samples and the ECMs. The observed outgrowth of the fruitbody‐producing ring of about 30 cm per year is a slightly higher growth rate than previously proposed for the Burgundy truffle, that is approximately 16 cm per year measured for mycelia in a mixed forest (Gryndler et al., 2015). As the fairy ring had a radius of about 4 m in 2017, this individual might then have been 13 years old. Population genetic analyses show that the genetic diversity is very low in this site, which corresponds well to the hypothesized population history to be probably founded by spores that originated from one or a few closely related fruitbodies that were deposited on rare occasions by dispersing animals. It is unclear why one individual outcompeted the others in growing and colonizing ECM root tips, but germinating, closely related spores of the opposite mating type could then act as paternal partners to form the fruitbodies.

The strong correlation between the amounts of soil mycelium, the presence of ECM root tips and the production of fruitbodies has been observed previously (Chen et al., 2021 and citations therein) and suggests that fruitbodies are preferentially formed in areas abundantly colonized by *T*. *aestivum* mycelium and where this species often dominates at fine root tips (for a more detailed discussion of mycelial quantities, see Supplementary Information). Interestingly, as reported for other fairy‐ring forming fungi (cf. Peter, 2006), the mycelium seems to decay inside the ring indicated by lower amounts of soil mycelium and the absence of ECMs within the ring. Nevertheless, we detected a patch of *T*. *aestivum* mycelium carrying the *MAT 1‐2* locus inside the ring spanned over five sampling positions. So far, no fruitbodies were harvested in this area but after a more thorough search for *T*. *aestivum* ECMs at this zone, we detected a new individual on several ECMs showing a *MAT 1‐2* genotype. This individual has neither acted as paternal nor maternal partner in the fruitbodies analysed so far and carries a new allele at the locus *aest18* that has not been detected in any other individuals at this site, but otherwise has the same alleles as the large maternal individual. It remains unclear whether spores carrying this allele were present since the first colonization event or whether this allele was introduced later. Assuming a growth rate of about 30 cm per year, this individual would be about 2 years old. It seems that once the fairy ring passed over that region and mycelia decomposed in the inside, which must have happened about 6–7 years ago, the mycelium of an opposite mating type was able to colonize roots and the soil. The young age of this perennial mycelium (i.e. 2 years old) might explain the absence of fruitbodies. Plantations of Burgundy truffle‐inoculated host trees require 5–10 years to produce fruitbodies (Stobbe et al., 2013). While in plantations a combination of settled soil conditions, the presence of spore inoculum for male partners as well as the ages of the tree and the mycelium may be decisive, in our sampling site, the first three mentioned conditions should be appropriate and only mycelial age varies compared to the productive maternal individual. It will be interesting to follow if this mycelial patch persists and grows, if and when fruitbodies will be formed, and whether these will structure themselves again on a new fairy ring.

Our study revealed that the Burgundy truffle has a similar life cycle to its better‐studied cousin, the Périgord truffle. In both species, generally few, long‐lived maternal mycelia produce most of the fruitbodies after mating with short‐lived paternal individuals that grow out from closely related ascospores. These findings provide a scientific foundation for the common practice in truffle farming of distributing spores under inoculated trees to increase productivity by providing paternal partners. Although rare, the ecological niche of perennial paternal individuals remains open and requires further research with intensive ECM sampling and monitoring of both the spatial distribution and timing of the presence of mating types in soils of productive sites. Furthermore, the presumed life cycle leading to severe inbreeding and restricted gene flow seen in natural Burgundy and Périgord truffle populations raises concerns about the adaptability of these economically and ecologically important ECM species to the rapid climate change we are already facing.

## CONFLICT OF INTEREST

The authors declare that they have no conflict of interest.

## Supporting information


**APPENDIX S1** Supporting Information

**APPENDIX S2** Supplementary Tables

## Data Availability

The data that support the findings of this study are available in the supplementary material of this article.

## References

[emi16131-bib-0001] Agerer, R. (1987‐2006) Colour atlas of Ectomycorrhizae. Schwäbisch Gmünd, DE: Einhorn Verlag.

[emi16131-bib-0002] Arnaud‐Haond, S. , Duarte, C.M. , Alberto, F. & SerrÃO, E.A. (2007) Standardizing methods to address clonality in population studies. Molecular Ecology Notes, 16, 5115–5139. 10.1111/j.1365-294X.2007.03535.x 17944846

[emi16131-bib-0003] Chen, J. , De la Varga, H. , Todesco, F. , Beacco, P. , Martino, E. , Le Tacon, F. et al. (2021) Frequency of the two mating types in the soil under productive and non‐productive trees in five French orchards of the Périgord black truffle (*Tuber melanosporum* Vittad.). Mycorrhiza, 31, 361–369. 33512580 10.1007/s00572-020-01011-4

[emi16131-bib-0004] De la Varga, H. , Le Tacon, F. , Lagoguet, M. , Todesco, F. , Varga, T. , Miquel, I. et al. (2017) Five years investigation of female and male genotypes in périgord black truffle (*Tuber melanosporum* Vittad.) revealed contrasted reproduction strategies. Environmental Microbiology, 19, 2604–2615. 28371112 10.1111/1462-2920.13735

[emi16131-bib-0005] Douhan, G.W. , Vincenot, L. , Gryta, H. & Selosse, M.‐A. (2011) Population genetics of ectomycorrhizal fungi: from current knowledge to emerging directions. Fungal Biology, 115, 569–597. 21724164 10.1016/j.funbio.2011.03.005

[emi16131-bib-0006] Dray, S. & Dufour, A.‐B. (2007) The ade4 package: implementing the duality diagram for ecologists. Journal of Statistical Software, 22, 1–20.

[emi16131-bib-0007] Earl, D.A. & von Holdt, B.M. (2012) STRUCTURE HARVESTER: a website and program for visualizing STRUCTURE output and implementing the Evanno method. Conservation Genetics Resources, 4, 359–361.

[emi16131-bib-0008] Evanno, G. , Regnaut, S. & Goudet, J. (2005) Detecting the number of clusters of individuals using the software structure: a simulation study. Molecular Ecology, 14, 2611–2620. 15969739 10.1111/j.1365-294X.2005.02553.x

[emi16131-bib-0009] Falush, D. , Stephens, M. & Pritchard, J.K. (2003) Inference of population structure using multilocus genotype data: linked loci and correlated allele frequencies. Genetics, 164, 1567–1587. 12930761 10.1093/genetics/164.4.1567 PMC1462648

[emi16131-bib-0010] Glass, N.L. , Jacobson, D.J. & Shiu, P.K.T. (2000) The genetics of hyphal fusion and vegetative incompatibility in filamentous ascomycete fungi. Annual Review of Genetics, 34, 165–186. 10.1146/annurev.genet.34.1.165 11092825

[emi16131-bib-0011] Gryndler, M. , Trilčová, J. , Hršelová, H. , Streiblová, E. , Gryndlerová, H. & Jansa, J. (2013) *Tuber aestivum* Vittad. mycelium quantified: advantages and limitations of a qPCR approach. Mycorrhiza, 23, 341–348. 23271632 10.1007/s00572-012-0475-6

[emi16131-bib-0012] Gryndler, M. , Beskid, O. , Hršelová, H. , Bukovská, P. , Hujslová, M. , Gryndlerová, H. et al. (2015) Mutabilis in mutabili: spatiotemporal dynamics of a truffle colony in soil. Soil Biology and Biochemistry, 90, 62–70.

[emi16131-bib-0013] Hardy, O.J. (2003) Estimation of pairwise relatedness between individuals and characterization of isolation‐by‐distance processes using dominant genetic markers. Molecular Ecology, 12, 1577–1588. 12755885 10.1046/j.1365-294x.2003.01835.x

[emi16131-bib-0014] Hardy, O.J. & Vekemans, X. (2002) spagedi: a versatile computer program to analyse spatial genetic structure at the individual or population levels. Molecular Ecology Notes, 2, 618–620.

[emi16131-bib-0015] Kopelman, N.M. , Mayzel, J. , Jakobsson, M. , Rosenberg, N.A. & Mayrose, I. (2015) Clumpak: a program for identifying clustering modes and packaging population structure inferences across K. Molecular Ecology Resources, 15, 1179–1191. 25684545 10.1111/1755-0998.12387 PMC4534335

[emi16131-bib-0016] Kronholm, I. , Loudet, O. & de Meaux, J. (2010) Influence of mutation rate on estimators of genetic differentiation ‐ lessons from *Arabidopsis thaliana* . BMC Genetics, 11, 33. 20433762 10.1186/1471-2156-11-33 PMC2888750

[emi16131-bib-0017] Le Tacon, F. , Zeller, B. , Plain, C. , Hossann, C. , Bréchet, C. & Robin, C. (2013) Carbon transfer from the host to *Tuber melanosporum* mycorrhizas and ascocarps followed using a ^13^C pulse‐labeling technique. PLoS One, 8, e64626. 23741356 10.1371/journal.pone.0064626 PMC3669392

[emi16131-bib-0018] Le Tacon, F. , Rubini, A. , Murat, C. , Riccioni, C. , Robin, C. , Belfiori, B. et al. (2016) Certainties and uncertainties about the life cycle of the Périgord black truffle (*Tuber melanosporum* Vittad.). Annals of Forest Science, 73, 105–117.

[emi16131-bib-0019] Leonardi, P. , Murat, C. , Puliga, F. , Iotti, M. & Zambonelli, A. (2019) Ascoma genotyping and mating type analyses of mycorrhizas and soil mycelia of *Tuber borchii* in a truffle orchard established by mycelial inoculated plants. Environmental Microbiology, 22, 964–975. 31393668 10.1111/1462-2920.14777

[emi16131-bib-0020] Loiselle, B.A. , Sork, V.L. , Nason, J. & Graham, C. (1995) Spatial genetic structure of a tropical understory shrub, *Psychotria officinalis* (Rubiaceae). American Journal of Botany, 82, 1420–1425.

[emi16131-bib-0021] Miyauchi, S. , Kiss, E. , Kuo, A. , Drula, E. , Kohler, A. , Sánchez‐García, M. et al. (2020) Large‐scale genome sequencing of mycorrhizal fungi provides insights into the early evolution of symbiotic traits. Nature Communications, 11, 5125. 10.1038/s41467-020-18795-w PMC7550596 33046698

[emi16131-bib-0022] Molinier, V. , Murat, C. , Morin, E. , Gollotte, A. , Wipf, D. & Martin, F. (2013) First identification of polymorphic microsatellite markers in the Burgundy Truffle, *Tuber aestivum* (Tuberaceae). Applications in Plant Science, 1, 1200220. 10.3732/apps.1200220 PMC4105367 25202513

[emi16131-bib-0023] Molinier, V. , Murat, C. , Frochot, H. , Wipf, D. & Splivallo, R. (2015a) Fine‐scale spatial genetic structure analysis of the black truffle *Tuber aestivum* and its link to aroma variability. Environmental Microbiology, 17, 3039–3050. 26036799 10.1111/1462-2920.12910

[emi16131-bib-0024] Molinier, V. , Murat, C. , Peter, M. , Gollotte, A. , De la Varga, H. , Meier, B. et al. (2015b) SSR‐based identification of genetic groups within European populations of *Tuber aestivum* Vittad. Mycorrhiza, 26, 99–110. 26070448 10.1007/s00572-015-0649-0

[emi16131-bib-0025] Molinier, V. , Peter, M. , Stobbe, U. & Egli, S. (2016a) The Burgundy truffle (*Tuber aestivum* syn. uncinatum): a truffle species with a wide habitat range over Europe. In: Zambonelli, A. , Iotti, M. & Murat, C. (Eds.) True truffle (Tuber spp.) in the world: soil ecology, systematics and biochemistry. Cham: Springer International Publishing, pp. 33–47.

[emi16131-bib-0026] Molinier, V. , Murat, C. , Baltensweiler, A. , Büntgen, U. , Martin, F. , Meier, B. et al. (2016b) Fine‐scale genetic structure of natural *Tuber aestivum* sites in southern Germany. Mycorrhiza, 26, 895–907. 27460217 10.1007/s00572-016-0719-y

[emi16131-bib-0027] Murat, C. , Rubini, A. , Riccioni, C. , De la Varga, H. , Akroume, E. , Belfiori, B. et al. (2013) Fine‐scale spatial genetic structure of the black truffle (*Tuber melanosporum*) investigated with neutral microsatellites and functional mating type genes. The New Phytologist, 199, 176–187. 23574460 10.1111/nph.12264

[emi16131-bib-0028] Murat, C. , Bonneau, L. , De La Varga, H. , Olivier, J.‐M. , Sandrine, F. & Le Tacon, F. (2016) Trapping truffle production in holes: a promising technique for improving production and unravelling truffle life cycle. Italian Journal of Mycology, 45, 47–53.

[emi16131-bib-0029] Murat, C. , Payen, T. , Noel, B. , Kuo, A. , Morin, E. , Chen, J. et al. (2018) Pezizomycetes genomes reveal the molecular basis of ectomycorrhizal truffle lifestyle. Nature Ecology and Evolution, 2, 1956–1965. 30420746 10.1038/s41559-018-0710-4

[emi16131-bib-0030] Paolocci, F. , Rubini, A. , Riccioni, C. & Arcioni, S. (2006) Reevaluation of the life cycle of *Tuber magnatum* . Applied and Environmental Microbiology, 72, 2390–2393. 16597935 10.1128/AEM.72.4.2390-2393.2006 PMC1449033

[emi16131-bib-0031] Peakall, R. & Smouse, P.E. (2012) GenAlEx 6.5: genetic analysis in excel. Population genetic software for teaching and research—an update. Bioinformatics, 28, 2537–2539. 22820204 10.1093/bioinformatics/bts460 PMC3463245

[emi16131-bib-0032] Peter, M. (2006) Ectomycorrhizal fungi ‐ fairy rings and the wood‐wide web. The New Phytologist, 171, 685–687. 16918540 10.1111/j.1469-8137.2006.01856.x

[emi16131-bib-0033] Pritchard, J.K. , Stephens, M. & Donnelly, P. (2000) Inference of population structure using multilocus genotype data. Genetics, 155, 945–959. 10835412 10.1093/genetics/155.2.945 PMC1461096

[emi16131-bib-0034] Reed, D.H. , Lowe, E.H. , Briscoe, D.A. & Frankham, R. (2003) Fitness and adaptation in a novel environment: effect of inbreeding, prior environment, and lineage. Evolution, 57, 1822–1828. 14503623 10.1111/j.0014-3820.2003.tb00589.x

[emi16131-bib-0035] Riccioni, C. , Belfiori, B. , Rubini, A. , Passeri, V. , Arcioni, S. & Paolocci, F. (2008) *Tuber melanosporum* outcrosses: analysis of the genetic diversity within and among its natural populations under this new scenario. The New Phytologist, 180, 466–478. 18643942 10.1111/j.1469-8137.2008.02560.x

[emi16131-bib-0036] Rubini, A. , Belfiori, B. , Riccioni, C. , Arcioni, S. , Martin, F. & Paolocci, F. (2011a) *Tuber melanosporum*: mating type distribution in a natural plantation and dynamics of strains of different mating types on the roots of nursery‐inoculated host plants. The New Phytologist, 189, 723–735. 20964691 10.1111/j.1469-8137.2010.03493.x

[emi16131-bib-0037] Rubini, A. , Belfiori, B. , Riccioni, C. , Tisserant, E. , Arcioni, S. , Martin, F. et al. (2011b) Isolation and characterization of MAT genes in the symbiotic ascomycete *Tuber melanosporum* . The New Phytologist, 189, 710–722. 20961294 10.1111/j.1469-8137.2010.03492.x

[emi16131-bib-0038] Schneider‐Maunoury, L. (2019) Écologie et biologie reproductive de la Truffe noire (*Tuber melanosporum* Vittad.). In: École Doctorale Sciences de la Nature et de l'Homme. Paris, F: Muséum national d'histoire naturelle, p. 202.

[emi16131-bib-0039] Schneider‐Maunoury, L. , Deveau, A. , Moreno, M. , Todesco, F. , Belmondo, S. , Murat, C. et al. (2020) Two ectomycorrhizal truffles, *Tuber melanosporum* and *T*. *aestivum*, endophytically colonise roots of non‐ectomycorrhizal plants in natural environments. The New Phytologist, 225, 2542–2556. 31733103 10.1111/nph.16321

[emi16131-bib-0040] Selosse, M.‐A. , Taschen, E. & Giraud, T. (2013) Do black truffles avoid sexual harassment by linking mating type and vegetative incompatibility? The New Phytologist, 199, 10–13. 23713552 10.1111/nph.12329

[emi16131-bib-0041] Selosse, M.‐A. , Schneider‐Maunoury, L. , Taschen, E. , Rousset, F. & Richard, F. (2017) Black truffle, a hermaphrodite with forced unisexual behaviour. Trends in Microbiology, 25, 784–787. 28622845 10.1016/j.tim.2017.05.010

[emi16131-bib-0042] Shiu, P.K. & Glass, N.L. (1999) Molecular characterization of *tol*, a mediator of mating‐type‐associated vegetative incompatibility in *Neurospora crassa* . Genetics, 151, 545–555. 9927450 10.1093/genetics/151.2.545 PMC1460514

[emi16131-bib-0043] Splivallo, R. , Vahdatzadeh, M. , Maciá‐Vicente, J.G. , Molinier, V. , Peter, M. , Egli, S. et al. (2019) Orchard conditions and fruiting body characteristics drive the microbiome of the black truffle *Tuber aestivum* . Frontiers in Microbiology, 10, 1437. 31316485 10.3389/fmicb.2019.01437 PMC6611097

[emi16131-bib-0044] Stenberg, P. , Lundmark, M. & Saura, A. (2003) mlgsim: a program for detecting clones using a simulation approach. Molecular Ecology Notes, 3, 329–331.

[emi16131-bib-0045] Stobbe, U. , Egli, S. , Tegel, W. , Peter, M. , Sproll, L. & Büntgen, U. (2013) Potential and limitations of Burgundy truffle cultivation. Applied Microbiology and Biotechnology, 97, 5215–5224. 23666478 10.1007/s00253-013-4956-0

[emi16131-bib-0046] Taschen, E. , Rousset, F. , Sauve, M. , Benoit, L. , Dubois, M.P. , Richard, F. et al. (2016) How the truffle got its mate: insights from genetic structure in spontaneous and planted Mediterranean populations of *Tuber melanosporum* . Molecular Ecology, 25, 5611–5627. 27717090 10.1111/mec.13864

[emi16131-bib-0047] Todesco, F. , Belmondo, S. , Guignet, Y. , Laurent, L. , Fizzala, S. , Tacon, F. et al. (2019) Soil temperature and hydric potential influences the monthly variations of soil *Tuber aestivum* DNA in a highly productive orchard. Scientific Reports, 9, 12964. 31506577 10.1038/s41598-019-49602-2 PMC6736833

[emi16131-bib-0048] Trappe, J. & Claridge, A. (2005) Hypogeous fungi: evolution of reproductive and dispersal strategies through interactions with animals and mycorrhizal plants. In: Dighton, J. , White, J.F. & Oudemans, P. (Eds.) The fungal community: its organization and role in the ecosystem. Boca Raton, FL: CRC Press, pp. 613–623.

[emi16131-bib-0049] Zambonelli, A. , Iotti, M. & Murat, C. (2016) True Truffle (Tuber spp.) In the world: soil ecology, systematics and biochemistry. Cham: Springer International Publishing.

