# Supplementary material for: Hidden fairy rings and males—Genetic patterns of natural Burgundy truffle (Tuber aestivum Vittad.) populations reveal new insights into its life cycle
Source: Environ Microbiol. 2022 Jul 20;24(12):6376–91. doi: 10.1111/1462-2920.16131 (PMC10084442; doi:10.1111/1462-2920.16131)
Supplement: Supplementary file 1 — APPENDIX S1 Supporting Information [file EMI-24-6376-s002.pdf]

Supplementary Information

Hidden fairy rings and males—Genetic patterns of natural Burgundy truffle (Tuber aestivum Vittad.) populations reveal new insights into its life cycle

Florian Staubli[1], Lea Imola[1], Benjamin Dauphin[1], Virginie Molinier[1], Stephanie Pfister[1], Yasmine Piñuela[1,2,3], Laura Schürz[1], Ludger Sproll[4], Brian S. Steidinger[1,5], Uli Stobbe[4], Willy Tegel[6], Ulf Büntgen[1,7,8,9], Simon Egli[1], Martina Peter[1*]

[1]*Swiss Federal Research Institute WSL, Birmensdorf, Switzerland*
[2]*Department of Crop and Forest Sciences, University of Lleida, Lleida, Spain*
[3]*Forest Science and Technology Centre of Catalonia, Solsona, Spain*
[4]*Deutsche Trüffelbäume, Bodman, Germany*
[5]*Department of Ecology, University of Konstanz, Konstanz, Germany*
[6]*Chair of Forest Growth, Albert-Ludwigs University, Freiburg, Germany*
[7]*Department of Geography, University of Cambridge, Cambridge, United Kingdom*
[8]*Global Change Research Centre (CzechGlobe), Brno, Czech Republic*
[9]*Department of Geography, Faculty of Science, Masaryk University, Brno, Czech Republic*

*Correspondence to: Martina Peter, martina.peter@wsl.ch

Supplementary Methods, Supplementary Discussion, Figs. S1- S12

Supplementary Methods:

Construction of a plasmid containing *T. aestivum* ITS sequence for standard curves in absolute qPCR quantification

To obtain a standard curve for absolute quantification of ITS copy numbers, a plasmid containing the *T. aestivum* ITS sequence was constructed as described in Supplementary Methods and Figures. For this, *T. aestivum* ITS sequence was amplified by PCR using the primers ITS1F and ITS4 (White *et al.*, 1990; Gardes and Bruns, 1993), the JumpStart REDTaq ReadyMix PCR Reaction Mix (Merck KGaA), and the same reaction mix as used for the PCR of mating type. PCR reactions were performed using an Applied Biosystems Veriti 96 Well Thermal Cycler (ThermoFisher Scientific) with the following conditions: 5 min at 95°C followed by 35 cycles of 30 sec denaturation at 95°C, 30 sec annealing at 56°C and 30 sec elongation at 72°C, with a final extension of 7 min at 72°C. The desired fragment was then cloned into a Mach1 T1 *E. coli* strain (Invitrogen, Basel, Switzerland) by using an Invitrogen TOPO TA Cloning Kit (ThermoFisher Scientific) with the protocol for the Mach1 T1 *E. coli* strain according to the manufacturer's instructions. The obtained strain was grown overnight at 37°C at 180 rpm in 4 ml of 50 μM Ampicillin (Duchefa Biochemie, Haarlem, The Netherlands) LB medium (Merck KGaA) and 3 ml of the overnight culture was extracted with a QIAprep Spin Miniprep Kit according to the manufacturer's instructions (Qiagen). Plasmid concentration was measured with a Qubit 3.0 Fluorometer (ThermoFisher Scientific) and a serial 1:10 dilution (10^7 to 10 plasmid copy numbers per μl) was performed based on the molecular weight of the plasmid.

Supplementary Discussion:

Mycelium quantities of Tuber aestivum in soil

When looking at the amounts of *T. aestivum* mycelium present in the soil of the WSL site, a maximum of 593 µg of dried soil mycelium per g of dried soil was quantified, which is slightly higher than that detected in a 2-year-old plantation in Spain (130 µg.g-1; Piñuela *et al.*, 2021), but lower than the amounts of 58,000 $\mu g.g^{-1}$ and 2,180 $\mu g.g^{-1}$ measured in a 12-year-old *T. aestivum* plantation in southwestern France (Todesco *et al.*, 2019) and in a natural forest in the Czech Republic (Gryndler *et al.*, 2013), respectively. Similarly high amounts of soil mycelium

were also found for *T. melanosporum* in a natural population (11,826 µg.g-1; Parladé *et al.*, 2013) and in truffle orchards under productive trees (94,000 µg.g-1; Chen *et al.*, 2021). Both black truffle species therefore seem to produce relatively dense soil mycelium as compared to other ECM species such as *Lactarius deliciosus* (86 µg.g-1; Parladé *et al.*, 2007), *T. magnatum* (98 µg.g-1; Iotti *et al.*, 2014) or *Boletus edulis* (950 µg.g-1; De la Varga *et al.*, 2012), although quantities likely vary depending on the season when samples are taken (Todesco *et al.*, 2019).

References

Chen, J., De la Varga, H., Todesco, F., Beacco, P., Martino, E., Le Tacon, F., and Murat, C. (2021) Frequency of the two mating types in the soil under productive and non-productive trees in five French orchards of the Périgord black truffle (*Tuber melanosporum* Vittad.). *Mycorrhiza* **31**: 361-369.

De la Varga, H., Águeda, B., Martínez-Peña, F., Parladé, J., and Pera, J. (2012) Quantification of extraradical soil mycelium and ectomycorrhizas of *Boletus edulis* in a Scots pine forest with variable sporocarp productivity. *Mycorrhiza* **22**: 59-68.

Gardes, M., and Bruns, T.D. (1993) ITS primers with enhanced specifity for basidiomycetes-application of the identification of mycorrhizae and rusts. *Mol Ecol* **2**: 113-118.

Gryndler, M., Trilčová, J., Hršelová, H., Streiblová, E., Gryndlerová, H., and Jansa, J. (2013) *Tuber aestivum* Vittad. mycelium quantified: advantages and limitations of a qPCR approach. *Mycorrhiza* **23**: 341-348.

Iotti, M., Leonardi, M., Lancellotti, E., Salerni, E., Oddis, M., Leonardi, P. *et al.* (2014) Spatio-temporal dynamic of *Tuber magnatum* mycelium in natural truffle grounds. *PLoS One* **9**: e115921.

Parladé, J., Hortal, S., Pera, J., and Galipienso, L. (2007) Quantitative detection of *Lactarius deliciosus* extraradical soil mycelium by real-time PCR and its application in the study of fungal persistence and interspecific competition. *J Biotechnol* **128**: 14-23.

Parladé, J., Varga, H., Miguel, A., Saez, R., and Pera, J. (2013) Quantification of extraradical mycelium of *Tuber melanosporum* in soils from truffle orchards in northern Spain. *Mycorrhiza* **23**: 99-106.

Piñuela, Y., Alday, J., Oliach, D., Castaño, C., Bolaño, F., Colinas, C., and Bonet, J. (2021) White mulch and irrigation increase black truffle soil mycelium when competing with summer truffle in young truffle orchards. Mycorrhiza 31: 371-382.

Todesco, F., Belmondo, S., Guignet, Y., Laurent, L., Fizzala, S., Tacon, F., and Murat, C. (2019) Soil temperature and hydric potential influences the monthly variations of soil *Tuber aestivum* DNA in a highly productive orchard. *Sci Rep* **9**.

White, T., Bruns, T., Lee, S., and Taylor, J. (1990) Amplification and direct sequencing of fungal ribosomal RNA genes for phylogenetics. In *PCR Protocols: a guide to methods and applications*. Innis, M.A., Gelfand, D.H., Sninsky, J.J., and White, T.J. (eds). New York, USA: Academic Press, pp. 315-322.

Supplementary Figures

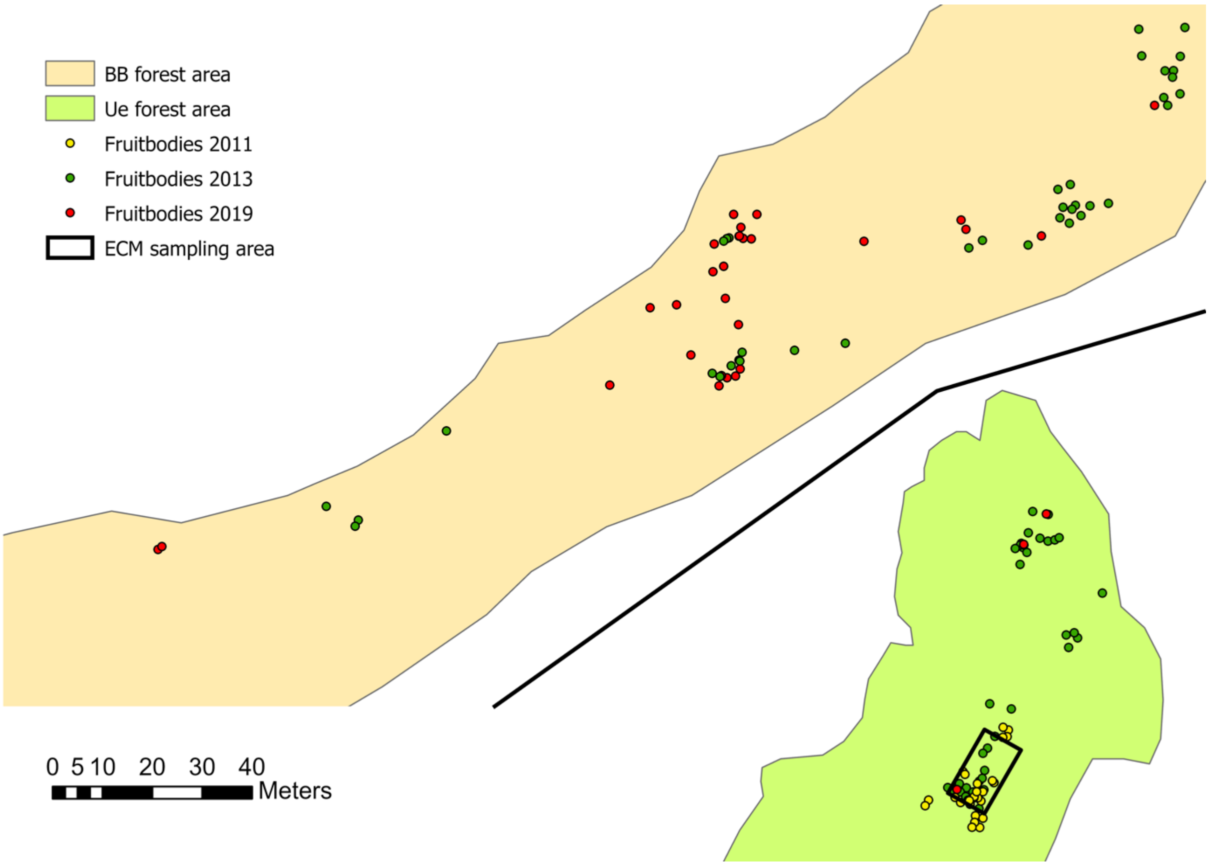

Fig. S1. Sampling overview of the BB and Ue sites. Fruitbodies harvested in different years are indicated by different colors and the ECM sampling area is given.

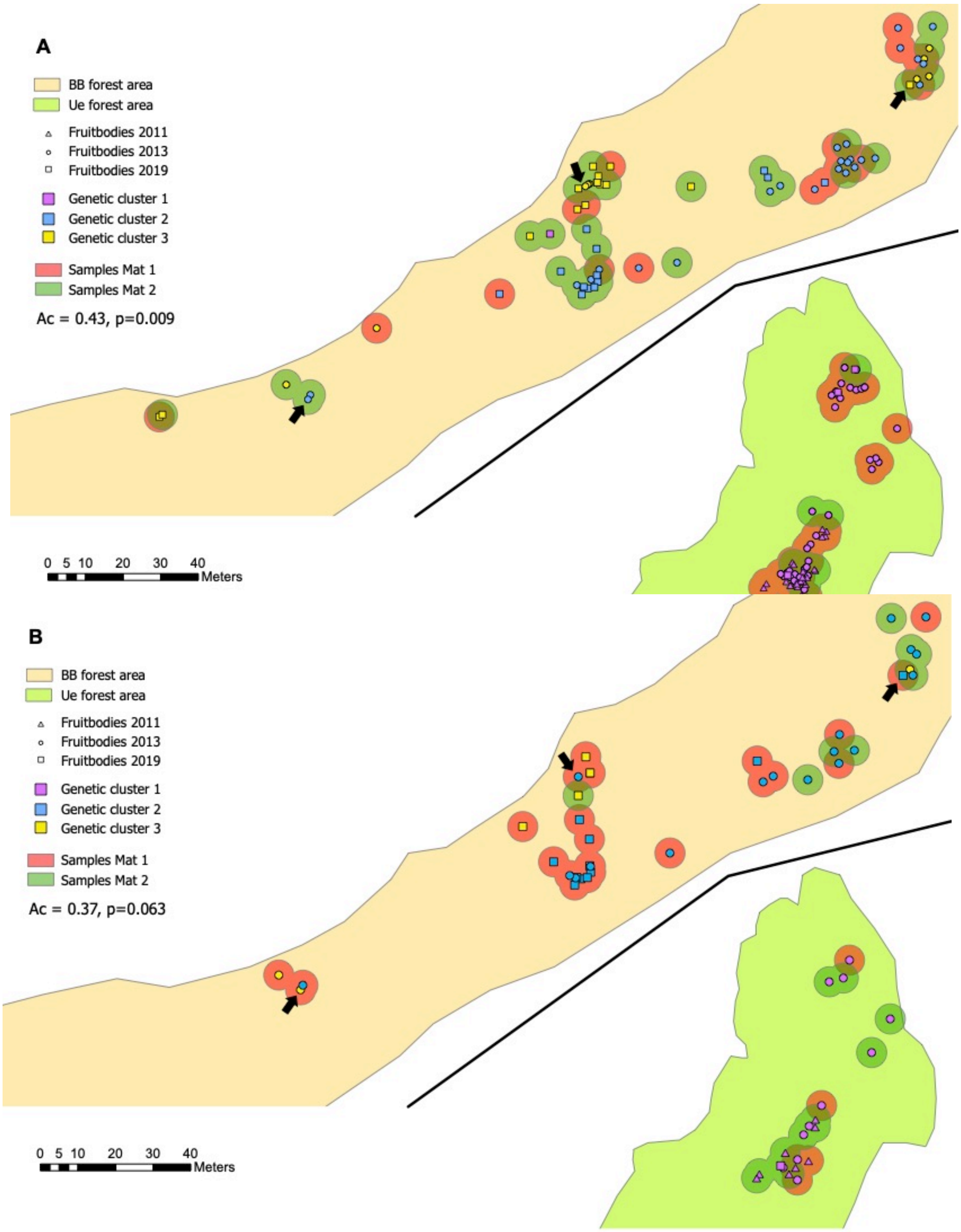

Fig. S2. Mating type distribution and genetic cluster membership of maternal (A) and paternal (B) individuals on the BB and Ue sites. Mating type and genetic clusters are indicated by different colors. The Aggregation Index (*Ac*) and corresponding *p* values for genetic cluster membership were calculated separately for both maternal and paternal individuals. Arrows indicate fruitbodies that were formed after mating of individuals from different genetic clusters.

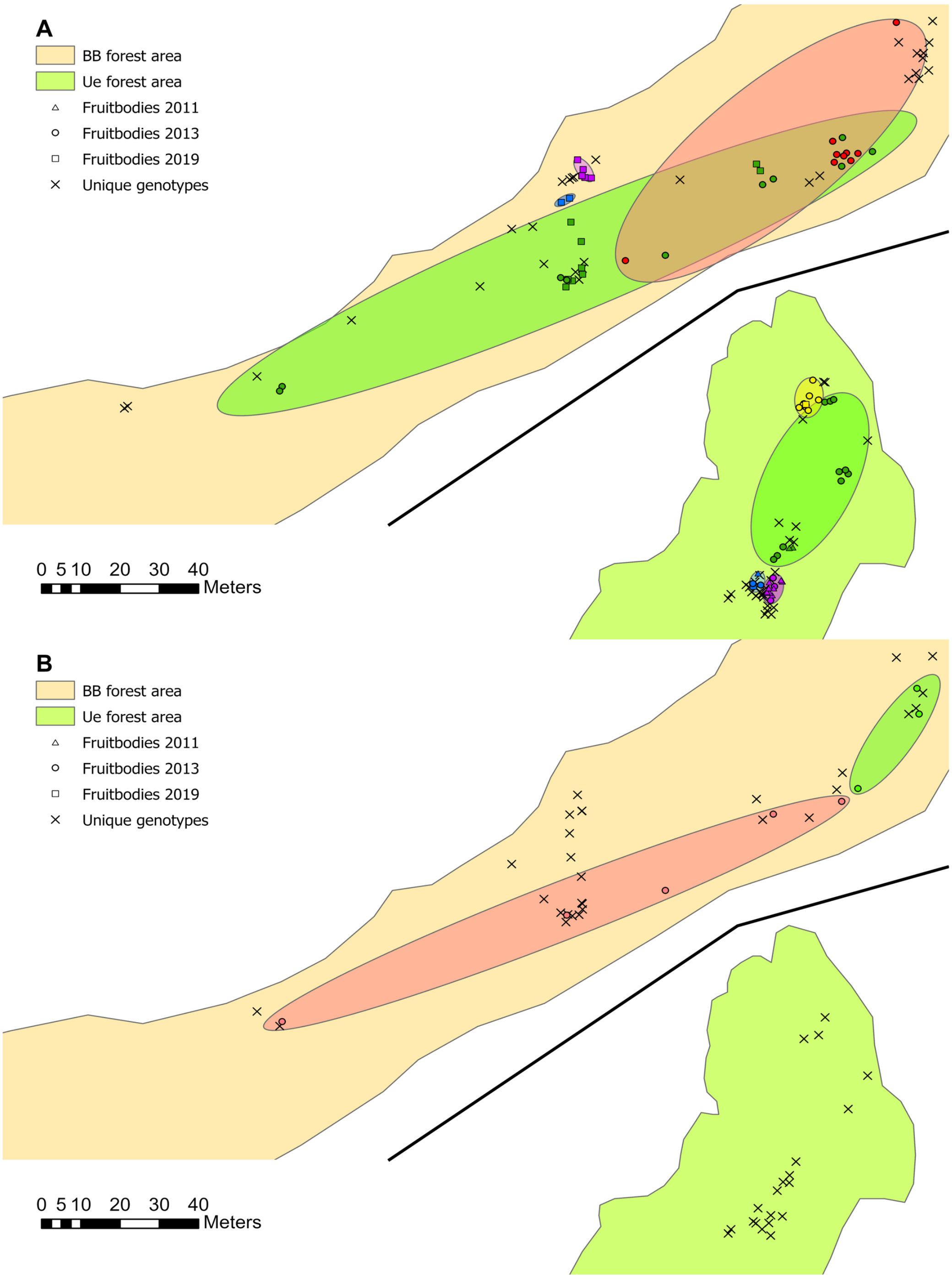

Fig. S3. MLG distribution of maternal (A) and paternal (B) individuals on the BB and Ue sites. Different MLGs are indicated by different colors and unique MLGs representing only one sample are marked by a cross.

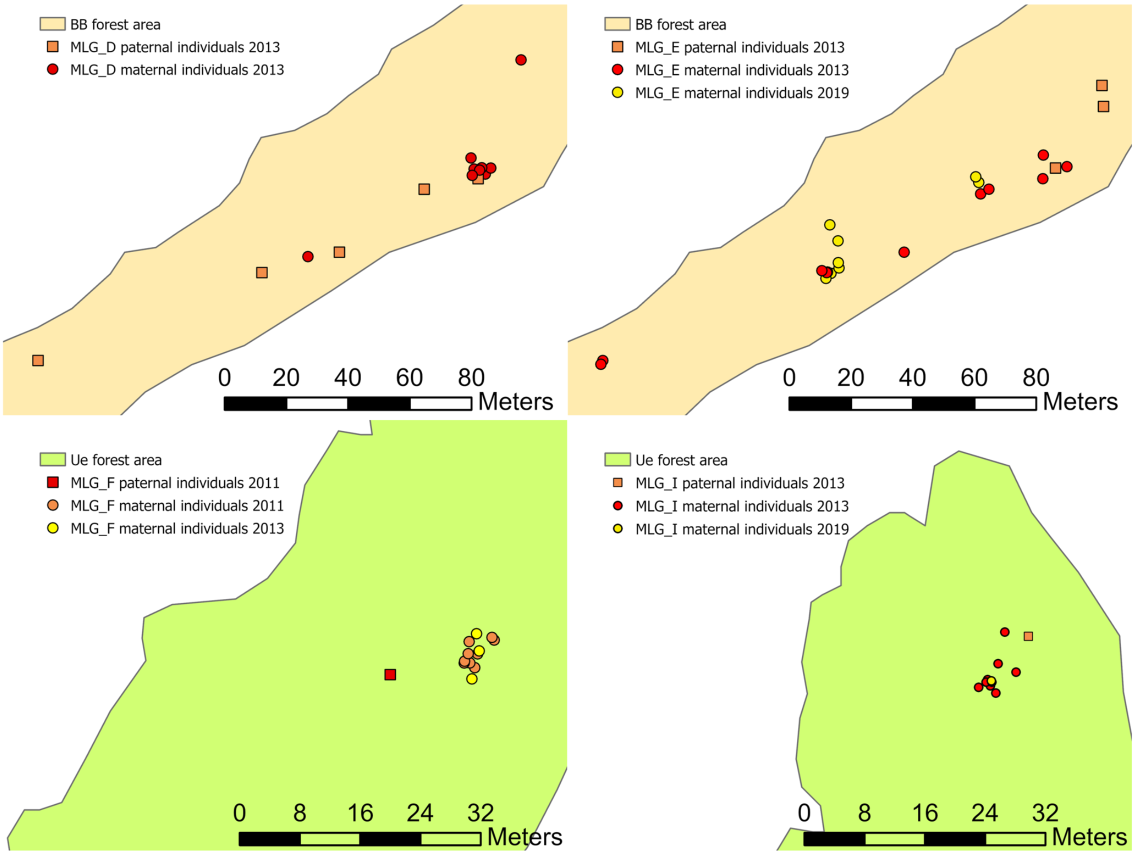

Fig. S4. Maps showing the distribution of maternal and paternal individuals of hermaphroditic MLGs with significant P_{sex} value. Maternal individuals are indicated by a circle and paternal ones by a square.

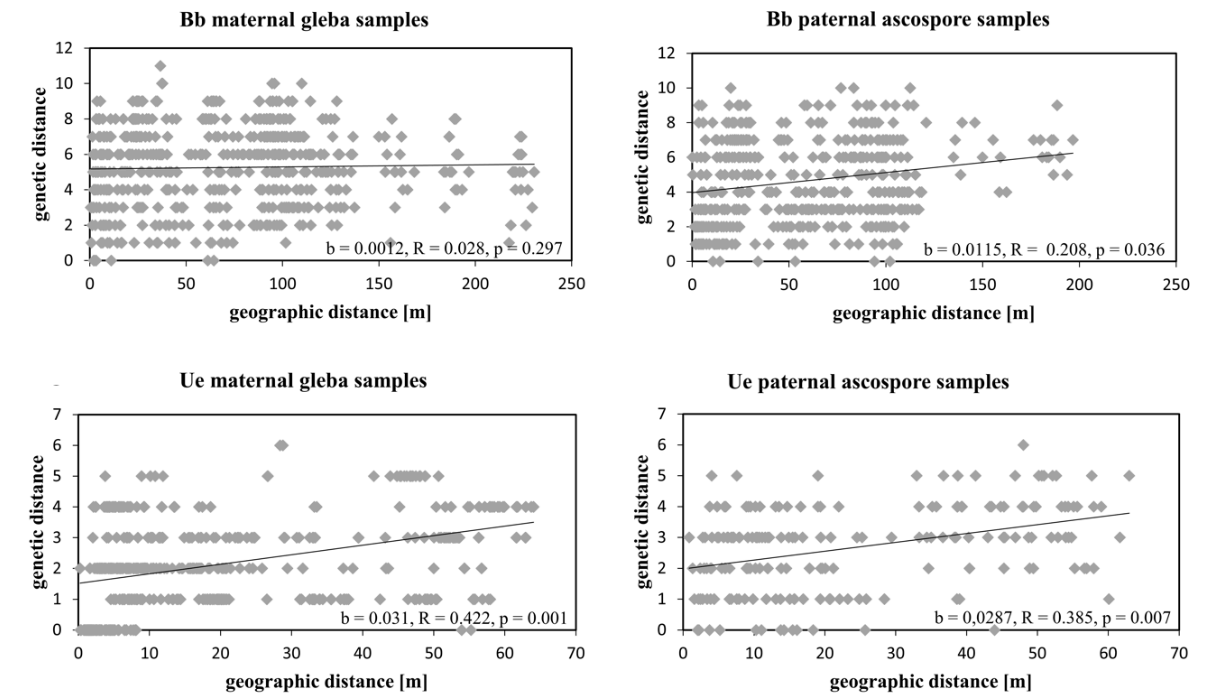

Fig. S5. Isolation by distance (IBD) analysis carried out on the clone-corrected datasets for maternal and paternal MLGs from the BB and Ue sites. IBD analysis, represented by the genetic versus geographic the distance, was carried out using a Mantel test based on 999 permutations. The slope value (*b*) of the linear regression, the Mantel coefficient of correlation (*R*) and the *p* value (*p*) are given.

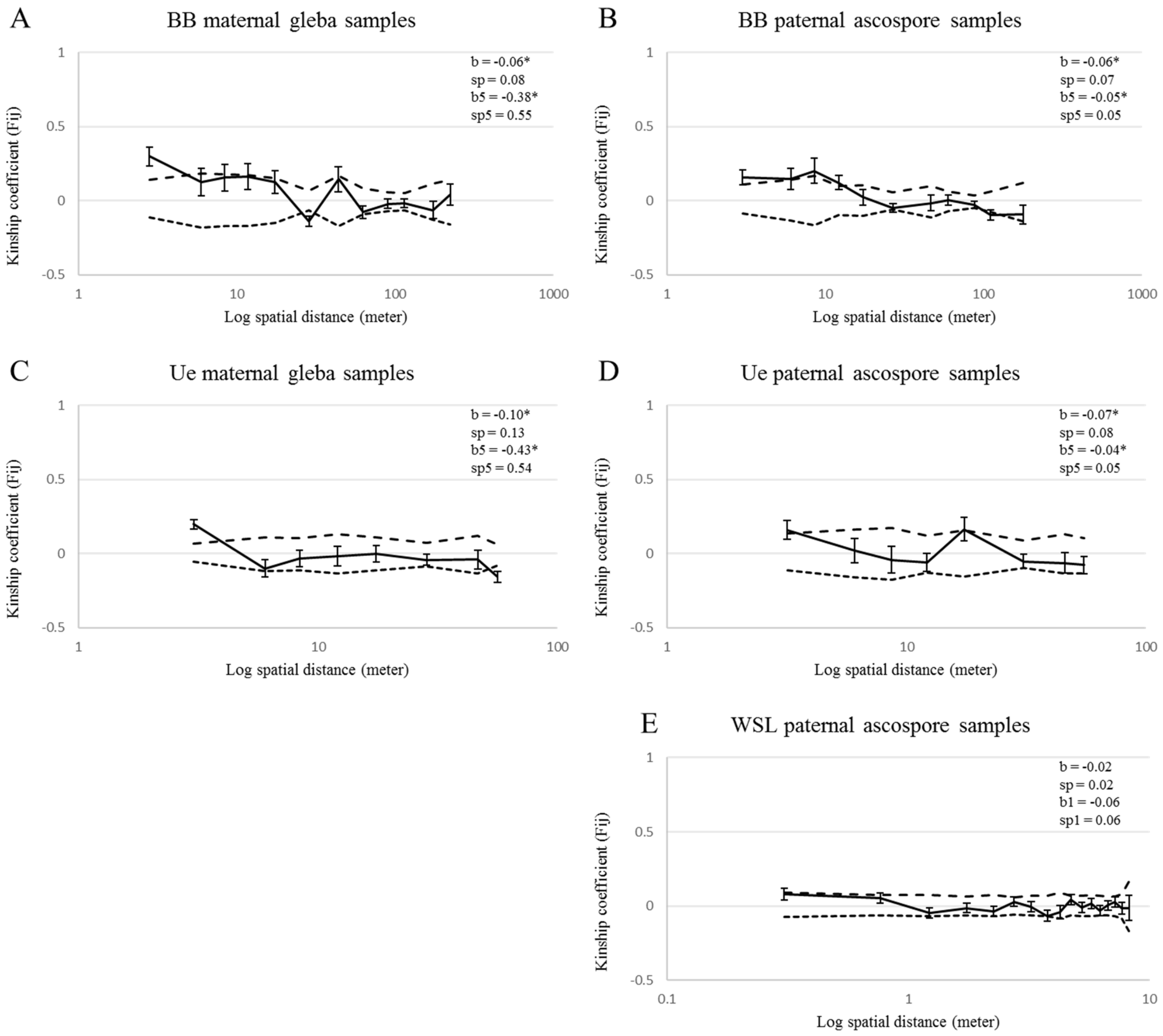

Fig. S6. Spatial auto-correlograms of the Kinship coefficient (F_{ij}) as a function of the logarithm of the geographic distance. Spatial auto-correlograms are given for clone-corrected datasets of maternal (A, C, E) and paternal (B, D, E) MLGs from BB (A, B), Ue (C, D), and WSL sites (E). 95% confidence intervals are indicated by dashed lines based on the null hypothesis of a random distribution of MLGs, calculated by 10,000 permutations of MLGs across individual geographic positions. The slope of the regression of kinship versus the natural logarithmic distance is given for all distance classes (*b*) and for pairs only within the first 5 m (b_5). *Sp* statistic defined as $–b / (1–F_1)$, where *b* represents the slope of regression and F_1 the mean F_{ij} value between pairs of the first distance class, is given for the whole distance range (*Sp*) and for the regression based on the first 5 m (Sp_5). Slope values with significant *p* values ($p < 0.05$) are indicated with an asterisk.

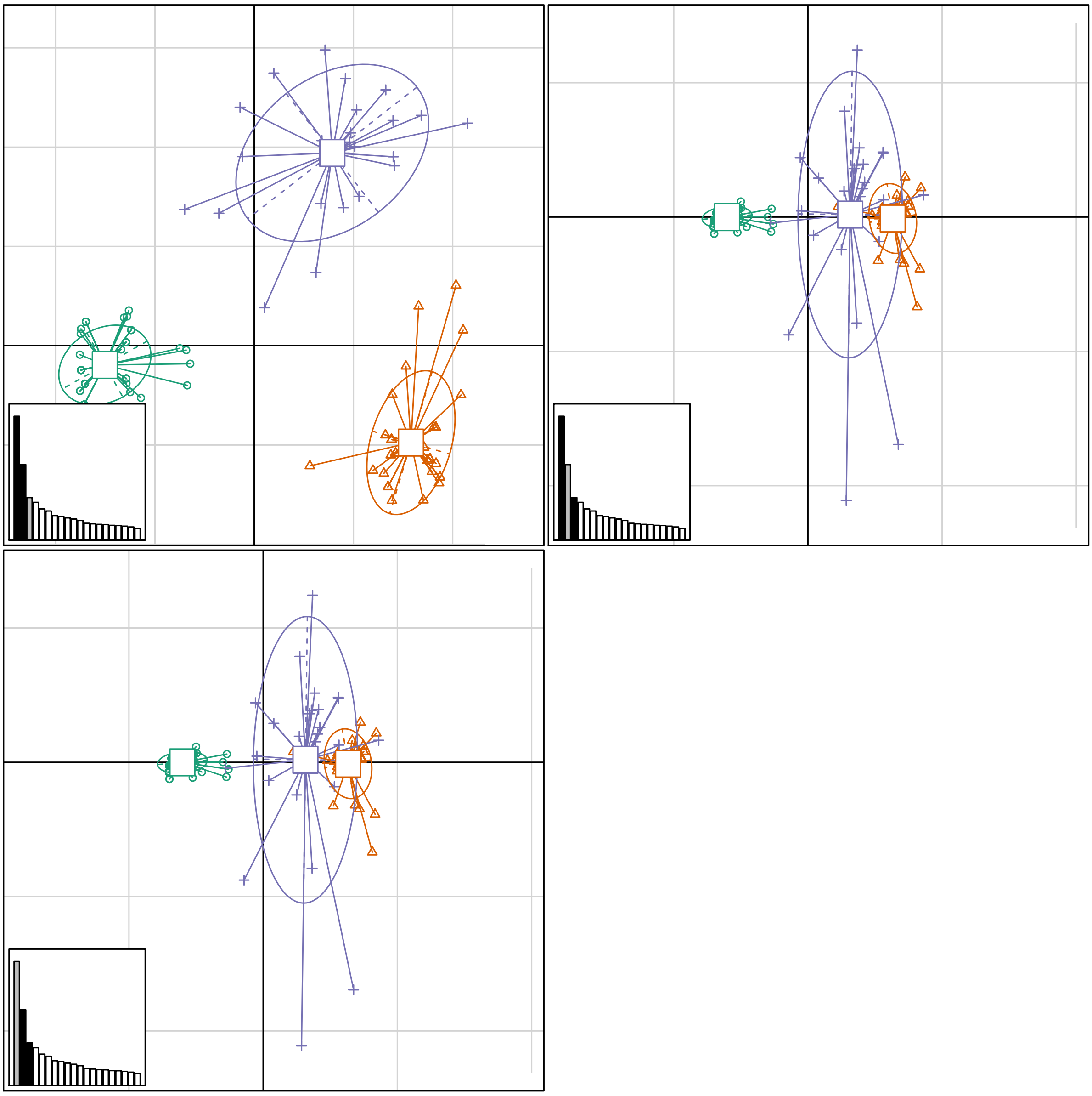

Fig. S7. Genetic structure of *Tuber aestivum* populations based on the subset clone-corrected dataset (populations Ue and BB). Principal component (PC) analysis of the subset clone-corrected dataset: A, PC1 and PC2, B, PC1 and PC3, and C, PC2 and PC3. Eigenvalues of PCs are shown in bottom left histograms. Colors and symbols indicate the genetic group of each individual as identified by ancestry coefficient scores.

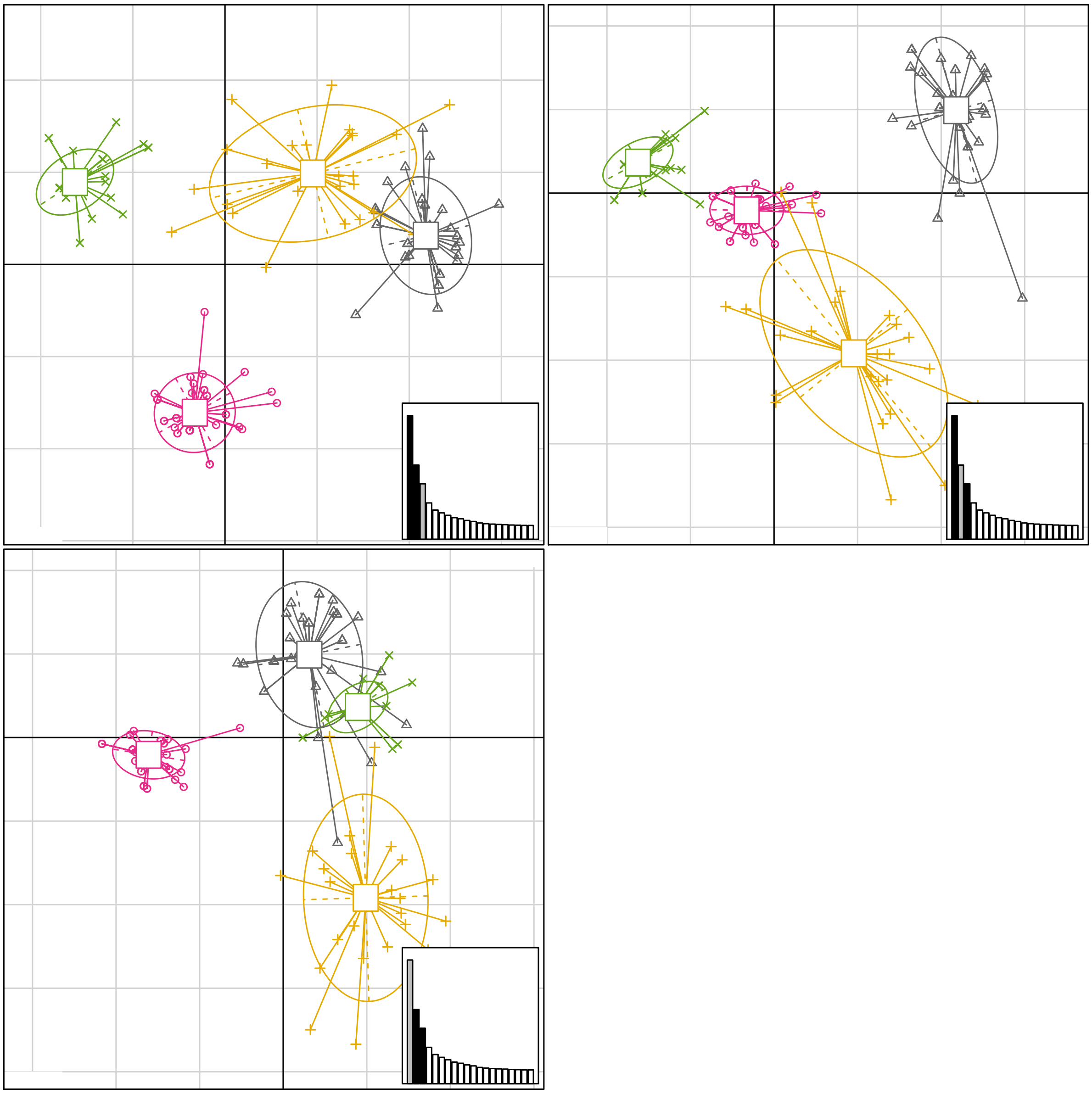

Fig. S8. Genetic structure of *Tuber aestivum* populations based on the complete clone-corrected dataset (all populations). Principal component (PC) analysis of the complete clone-corrected dataset: A, PC1 and PC2, B, PC1 and PC3, and C, PC2 and PC3. Eigenvalues of PCs are shown in bottom right histograms. Colors and symbols indicate the genetic group of each individual as identified by ancestry coefficient scores.

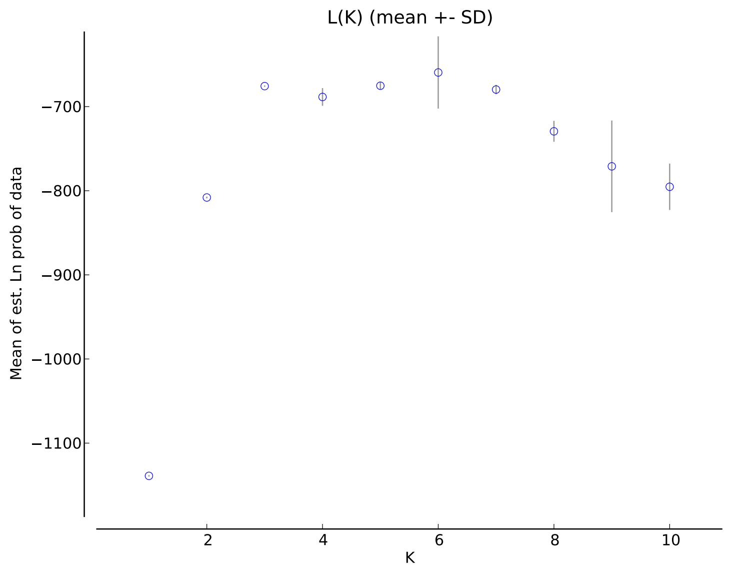

Fig. S9. Distribution of estimated natural logarithm probability of the subset clone-corrected dataset (sites from southern Germany) across *K* values in Bayesian clustering analysis. Mean and standard deviation of each *K* value were assessed from ten replicates.

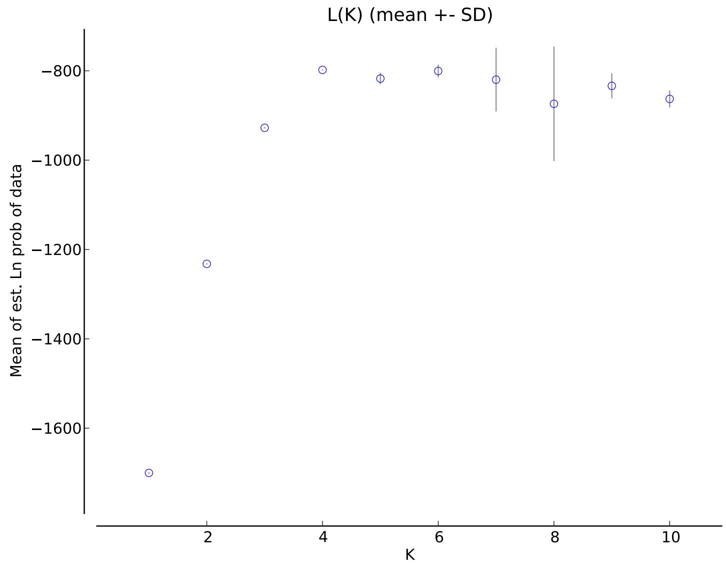

Fig. S10. Distribution of estimated natural logarithm probability of the complete clone-corrected dataset (BB, Ue, and WSL sites) across *K* values in Bayesian clustering analysis. Mean and standard deviation of each *K* value were assessed from ten replicates.

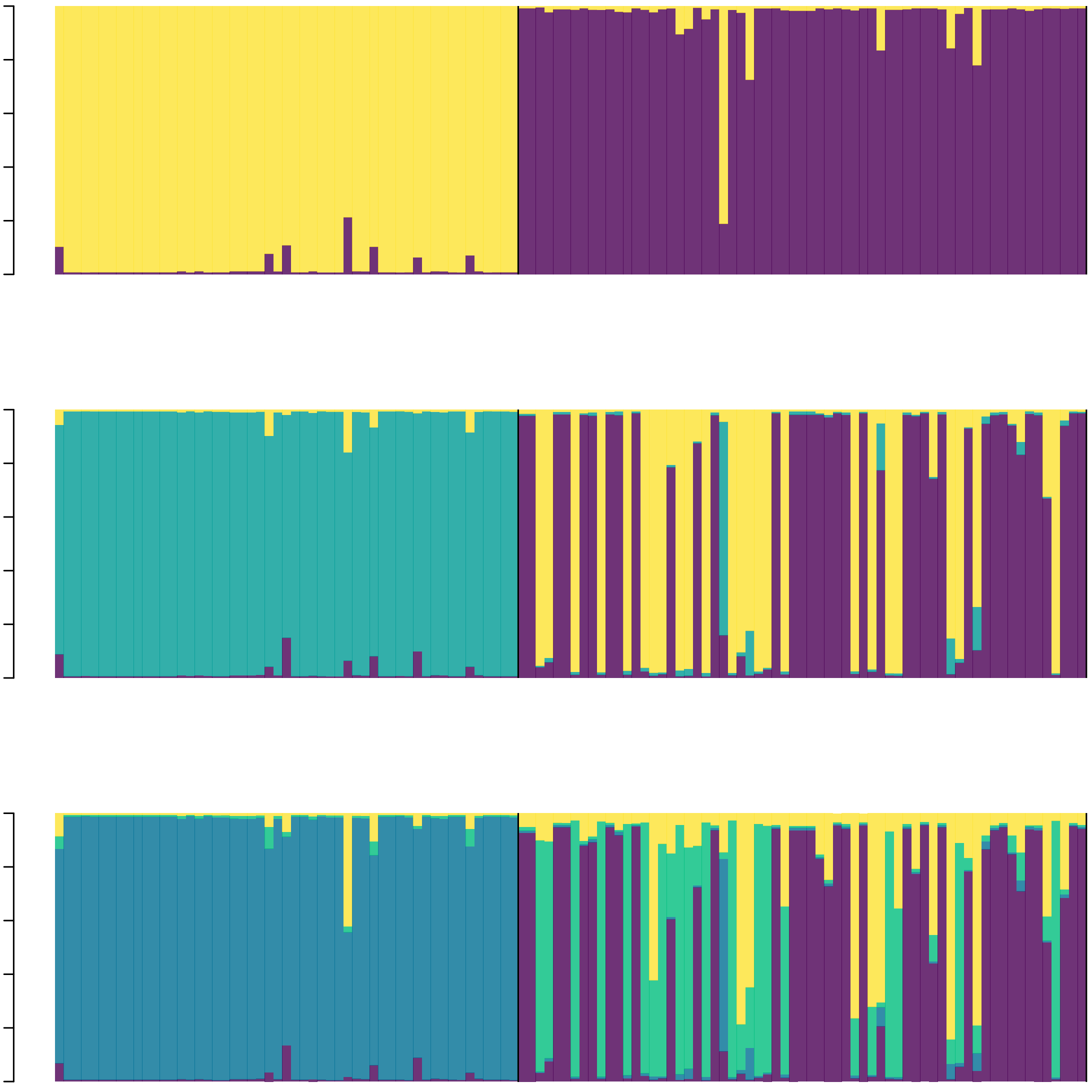

Fig. S11. Admixture analysis among the two southern Germany sites of *Tuber aestivum* based on the subset clone-corrected dataset for K = 2–3. Each barplot shows the level of admixture within individuals and colors reflect assignment probabilities to respective genetic group.

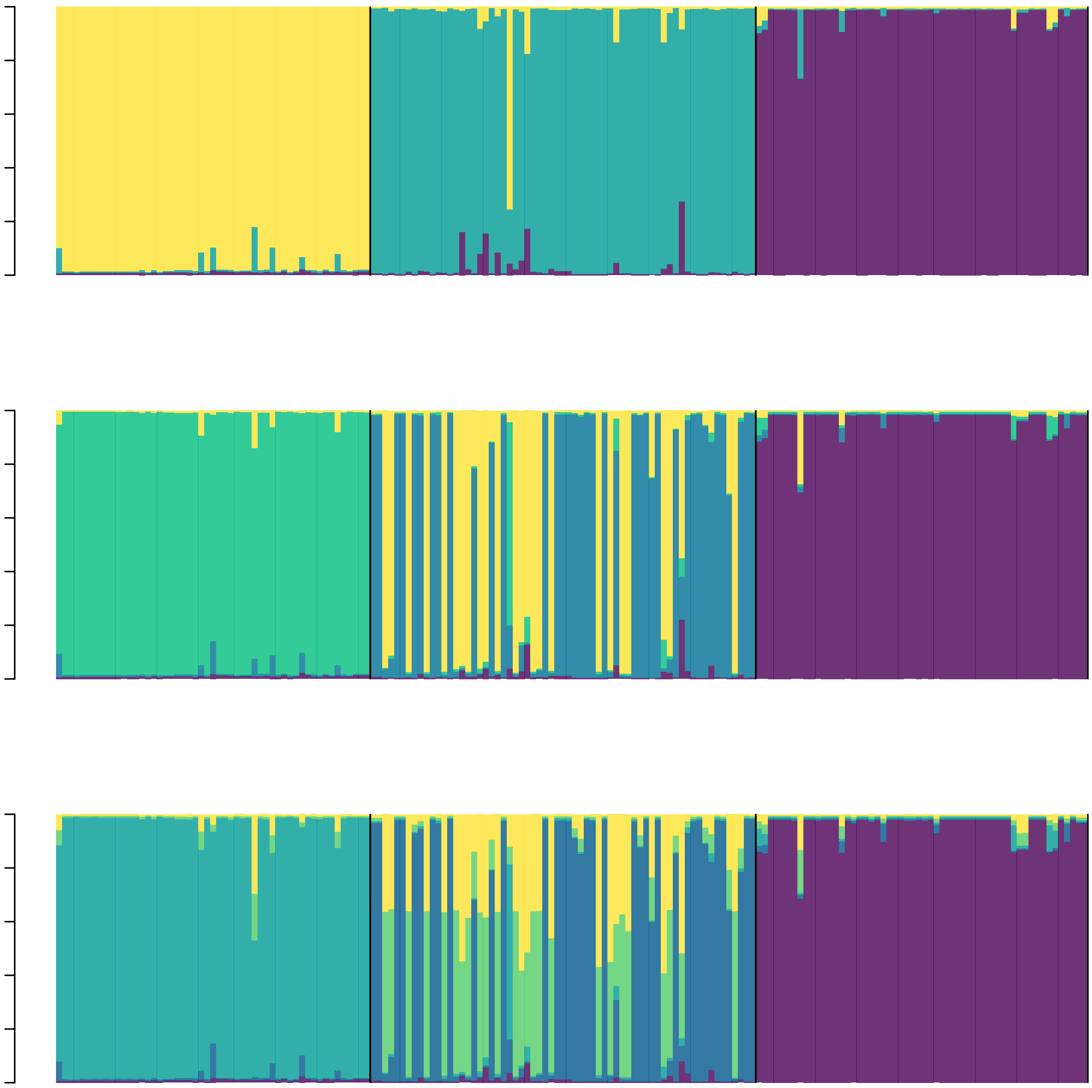

Fig. S12. Admixture analysis among the three sites BB, Ue, and WSL of *Tuber aestivum* based on the complete clone-corrected dataset for $K = 3–5$. Each barplot shows the level of admixture within individuals and colors reflect assignment probabilities to respective genetic group.
